# STK25 inhibits cancer‐associated fibroblast activation to overcome cetuximab resistance in colorectal cancer

**DOI:** 10.1002/ctm2.70678

**Published:** 2026-04-29

**Authors:** Yifan Hou, Jiangbo Chen, Hao Hao, Tongkun Song, Lin Song, Pu Xing, Kai Weng, Yumeng Ran, Xinying Yang, Xiaowen Qiao, Jie Chen, Ruibin Yao, Hong Yang, Lei Chen, Jiabo Di, Kai Xu, Xiangqian Su, Beihai Jiang

**Affiliations:** ^1^ Key Laboratory of Carcinogenesis and Translational Research (Ministry of Education), Department of Gastrointestinal Surgery IV Peking University Cancer Hospital & Institute Beijing China; ^2^ Department of Education Peking University Third Hospital Beijing China; ^3^ Department of Clinical Medicine Capital Medical University Beijing China; ^4^ Department of Gastrointestinal Surgery Peking University Cancer Hospital (Inner Mongolia Campus) & Affiliated Cancer Hospital of Inner Mongolia Medical University Hohhot China; ^5^ State Key Laboratory of Holistic Integrative Management of Gastrointestinal Cancers, Department of Gastrointestinal Surgery IV Peking University Cancer Hospital & Institute Beijing China

**Keywords:** AREG/EGFR axis, cancer‐associated fibroblasts, cetuximab resistance, colorectal cancer, NF‐κB, STK25

## Abstract

**Background:**

Cancer‐associated fibroblasts (CAFs) within the tumour microenvironment play a pivotal role in colorectal cancer (CRC) progression and therapeutic resistance. Serine/threonine protein kinase 25 (STK25) exerts multiple roles in tumourigenesis; however, its role in mediating tumour–stroma crosstalk remains largely unexplored.

**Methods:**

Primary CAFs were isolated from CRC patient tissues and characterised to confirm their identity. The effects of STK25 expression on CAF activity and CAF‐mediated tumour progression were evaluated both in vitro and in vivo. Western blot, qRT‐PCR, ChIP and dual‐luciferase reporter assays were performed to elucidate the mechanism by which STK25 regulated CAF activation. Moreover, the effect of STK25 expression on CAF‐induced cetuximab resistance was assessed in vitro and in vivo. The clinical significance of STK25 expression was determined in CRC patient tissues, tissue microarrays and patient‐derived organoids.

**Results:**

Knockdown of STK25 in CRC cells enhanced CAF proliferation, migration and activation, whereas its overexpression exhibited the opposite effect. STK25‐knockdown‐mediated CAF activation subsequently promoted CRC cell proliferation and metastasis. Moreover, STK25 depletion combined with CAFs significantly enhanced CRC tumour growth in vivo. Mechanistically, STK25 deficiency activated the NF‐κB pathway, leading to p50 phosphorylation which directly bound to the AREG promoter, thereby transcriptionally up‐regulating AREG expression. In addition, STK25‐regulated AREG/EGFR axis mediated the crosstalk between CRC cells and CAFs. More importantly, CAFs conferred resistance to the anti‐EGFR antibody cetuximab, which could be reversed either by STK25 overexpression or by AREG‐neutralising antibody treatment. Clinically, low STK25 expression correlated with elevated CAFs marker levels and poor cetuximab response in CRC patients.

**Conclusions:**

Our findings identified STK25 as a critical regulator of the NF‐κB/AREG/EGFR axis in tumour–CAF communication and highlight its potential as a therapeutic target for overcoming CAF‐induced cetuximab resistance in CRC.

**Key points:**

- STK25 deficiency enhanced CAF‐mediated CRC growth via the NF‐κB/AREG/EGFR axis.
- STK25 overexpression or AREG antibody overcame CAF‐mediated cetuximab resistance.
- CRC patients with high STK25 and low CAFs marker levels might benefit from cetuximab treatment.

## Graphical abstract

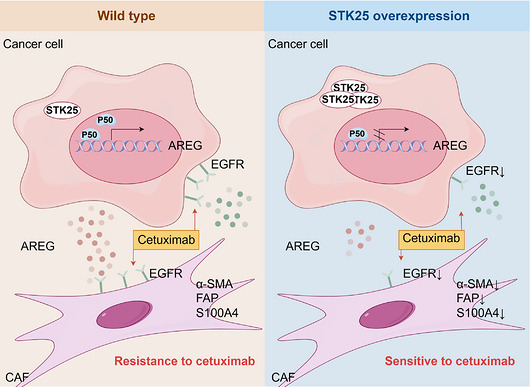

## 1 INTRODUCTION

Colorectal cancer (CRC) remains one of the leading causes of global cancer incidence and mortality.^1^, ^2^ The tumour microenvironment (TME) contributes substantially to the tumour progression. Among its components, cancer‐associated fibroblasts (CAFs) constitute the predominant stromal cell population in solid tumours and play critical roles in supporting tumourigenesis and modulating drug resistance. Therefore, elucidating the mechanisms underlying the crosstalk between tumour cells and CAFs in CRC has significant clinical potential for the development of novel targeted therapeutic strategies.

CAFs promote tumour invasion and metastasis through the secretion of paracrine factors, direct cell–cell interactions and extracellular matrix (ECM) remodelling. In the TME of hepatocellular carcinoma (HCC), tumour‐derived SPP1 promoted the differentiation of hepatic stellate cells into CAFs, thereby contributing to hepatic fibrogenesis and tumour progression.^3^ Wang et al. identified that tumour‐derived CCL15 facilitated communication between HCC cells and CAFs to promote HCC growth.^4^ Furthermore, IL‐6 secreted by CAFs up‐regulated LRG1 expression in CRC cells by activating the JAK2/STAT3 signalling pathway, which in turn enhances tumour invasion and metastasis.^5^

Activation of the NF‐κB pathway is known to promote tumour growth and progression, as well as being associated with the modulation of CAF activation and infiltration in TME.^6^, ^7^, ^8^, ^9^ Overexpression of COL11A1 in ovarian cancer cells induced CAF activation by regulating TGF‐β3 through the NF‐κB/IGFBP2 signalling axis.^7^ In gastric cancer, Helicobacter pylori‐induced NF‐κB activation stimulated the PIEZO1–YAP1–CTGF signalling cascade, thereby facilitating TME remodelling through enhanced CAF infiltration.^8^ Wang et al. found that EMP1 depletion in triple‐negative breast cancer (TNBC) cells reduced IL6 secretion via the NF‐κB pathway, which inhibited CAF proliferation and subsequently suppressed TNBC progression.^9^ Therefore, a comprehensive understanding of cancer cell–CAF communication‐mediated NF‐κB signalling may provide new insights into the development of effective cancer therapies.

The epidermal growth factor receptor (EGFR)‐targeted blockade therapies are well established in clinical practice, including the monoclonal antibodies cetuximab and panitumumab for patients with RAS wild‐type metastatic CRC (mCRC), as well as the tyrosine kinase inhibitor gefitinib for those with non‐small cell lung cancer (NSCLC) harbouring EGFR mutations. Recent studies have revealed that CAF activation contributed to tumour cell resistance to the EGFR‐targeted therapies.^10^, ^11^, ^12^, ^13^, ^14^ As the ligand of EGFR, amphiregulin (AREG) is frequently up‐regulated in tumour tissues and has been implicated in fibroblast activation and proliferation.^15^ Therefore, investigating the regulatory mechanism underlying the AREG/EGFR axis in tumour cell–CAF interactions may offer new strategies to overcome resistance to EGFR‐targeted therapies in CRC.

Serine/threonine protein kinase 25 (STK25), also known as SOK1 or YSK1, plays critical roles in multiple biological processes, including lipid and glucose metabolism,^16^, ^17^, ^18^ inflammatory response,^19^ cell polarisation and migration, cell death and tumour progression.^20^, ^21^, ^22^, ^23^, ^24^, ^25^ STK25 deficiency enhances systemic glucose tolerance and increased insulin sensitivity,^16^ indicating that STK25 may serve as a potential therapeutic target for type 2 diabetes. STK25 could mediate inflammation through TLR‐induced activation of IRF5 in immune cells.^19^ Previous study has demonstrated that STK25 regulated Golgi morphology and involved in neuronal polarisation and migration. In addition, STK25 expression promotes apoptotic cell death following chemical anoxia, which depends on its translocation from the Golgi apparatus to the nucleus.^25^

The role of STK25 in different tumour types remains controversial. STK25 exhibited elevated expression and promoted tumour progression in prostate cancer, HCC and CRC. However, STK25 functioned as a tumour suppressor in neuroblastoma, pancreatic ductal adenocarcinoma (PDAC), breast cancer and CRC.^25^, ^26^ Our previous studies showed that high STK25 levels inhibited cell proliferation by attenuating glycolytic activity, whereas loss of STK25 induced autophagic processes through activation of the JAK2/STAT3 pathway.^20^, ^21^ Additionally, our previous study revealed that STK25 deficiency up‐regulated PD‐L1 expression to inhibit the anti‐tumour immune response and facilitate the sensitivity of CRC to immune checkpoint blockade (ICB) therapy.^27^ Nevertheless, the role of STK25 in regulating stromal components, particularly CAFs, within the TME remains largely unexplored.

In this study, we found the crucial role of STK25 in CAF‐mediated CRC progression and cetuximab resistance. We demonstrated that STK25 depletion in CRC cells promoted CAF activation, which facilitated CRC progression through the AREG/EGFR signalling axis. Mechanistically, STK25 regulated AREG expression at the transcriptional level in an NF‐κB‐dependent manner. Moreover, up‐regulation of STK25 or anti‐AREG antibody treatment effectively overcame CAF‐induced cetuximab resistance in CRC cells. Collectively, our findings highlighted STK25 as a promising therapeutic target and provided potential strategies to overcome cetuximab resistance in CRC patients.

## 2 MATERIALS AND METHODS

### 2.1 Patients and tissue samples

A human CRC tissue microarray containing 60 patient samples was obtained from Shanghai Outdo Biotech. Paired CRC specimens and normal tissues were collected from the Department of Gastrointestinal Surgery IV at Peking University Cancer Hospital & Institute for western blot (Table S1) and qRT‐PCR (Table S2) analysis. Additionally, tumour samples prepared for immunohistochemistry (IHC) were obtained from cetuximab‐sensitive and cetuximab‐resistant CRC patients (Table S3). Additionally, tumour tissues for organoid establishment were obtained from CRC patients who underwent surgical resection without neoadjuvant therapy (Table S4). Comprehensive clinicopathological information, including TNM stage and histological subtype, was available for all enrolled patients. All experimental procedures were conducted in accordance with the Declaration of Helsinki and were approved by the Research Ethics Committee of Peking University Cancer Hospital & Institute (2021KT136), and written informed consent was obtained from all patients.

### 2.2 Primary fibroblast isolation

Primary CAFs and normal fibroblasts (NFs) were isolated from the tumour and matched adjacent normal tissues derived from patients without receiving neoadjuvant therapy. Briefly, tissues were dissected into pieces of approximately 2 mm^2^, and tissue fragments were maintained in 10 cm culture dishes for approximately 4 weeks with medium renewed every 2 days until fibroblasts migrated out from the tissue pieces. Cells were then harvested using trypsin (LVN1006; Livning, China) and expanded for experiments within six passages.

### 2.3 In vivo tumourigenesis assays

Animal studies were approved by the Ethics Committee of Peking University Cancer Hospital & Institute (2021KT134). To establish subcutaneous xenografts, 4‐week‐old male BALB/c nude mice were randomly allocated into four groups and subcutaneously injected in the left flank with either indicated RKO cells alone (2 × 10^6^) or in combination with CAFs (1 × 10^6^). Four experimental groups were established: sgCtrl–RKO, sgSTK25–RKO, sgCtrl–RKO + CAFs and sgSTK25–RKO + CAFs (*n* = 8 per group). Tumour growth was monitored at regular intervals, and volume was calculated using the formula *V* = .5 × (short diameter)^2^ × long diameter. After 36 days, mice were sacrificed, and tumours were collected for measurement, weighing and histological evaluation by IHC.

To investigate the sensitivity of cetuximab in vivo, nude mice subcutaneously injected with sgSTK25–RKO cells mixed with CAFs were intraperitoneally injected with 1 mg cetuximab or IgG (*n* = 6 per group). Mice were sacrificed on day 40. Statistical analyses of mice tumour volumes were performed using one‐way ANOVA and two‐way ANOVA.

### 2.4 Statistical analysis

Statistical analyses were conducted using GraphPad Prism 9.5. For comparisons between two independent groups, an unpaired two‐tailed Student's *t*‐test was utilised. For multiple group comparisons, one‐way ANOVA or two‐way ANOVA was applied as appropriate. All experiments were independently repeated at least three times, with results shown as mean ± SD. A *p* value < .05 was considered statistically significant.

### 2.5 Additional experimental procedures

All other experimental procedures are fully described in the Supporting Information: Materials and Methods.

## 3 RESULTS

### 3.1 STK25 depletion in CRC cells promoted the proliferation, migration and activation of CAFs

To determine the effects of STK25 in CRC cells on the regulation of CAF activation, fibroblasts were isolated from tumour tissues (designated as CAFs) and adjacent normal tissues (designated as NFs) of CRC patients (Figure S1A). Western blot assays were performed to characterise the molecular phenotype of NFs and CAFs. The results revealed that the expression of general markers of activated fibroblasts, including fibroblast activation protein (FAP), α‐smooth muscle actin (α‐SMA), vimentin and S100 calcium‐binding protein A4 (S100A4), were markedly up‐regulated in CAFs compared with NFs (Figure S1B). Additionally, RKO and LoVo cells cultured in conditioned medium (CM) from CAFs (CAF‐CM) exhibited increased cell proliferation and migration, compared with CRC cells cultured in CM from NFs (NF‐CM) (Figure S1C–E).

**FIGURE 1:**
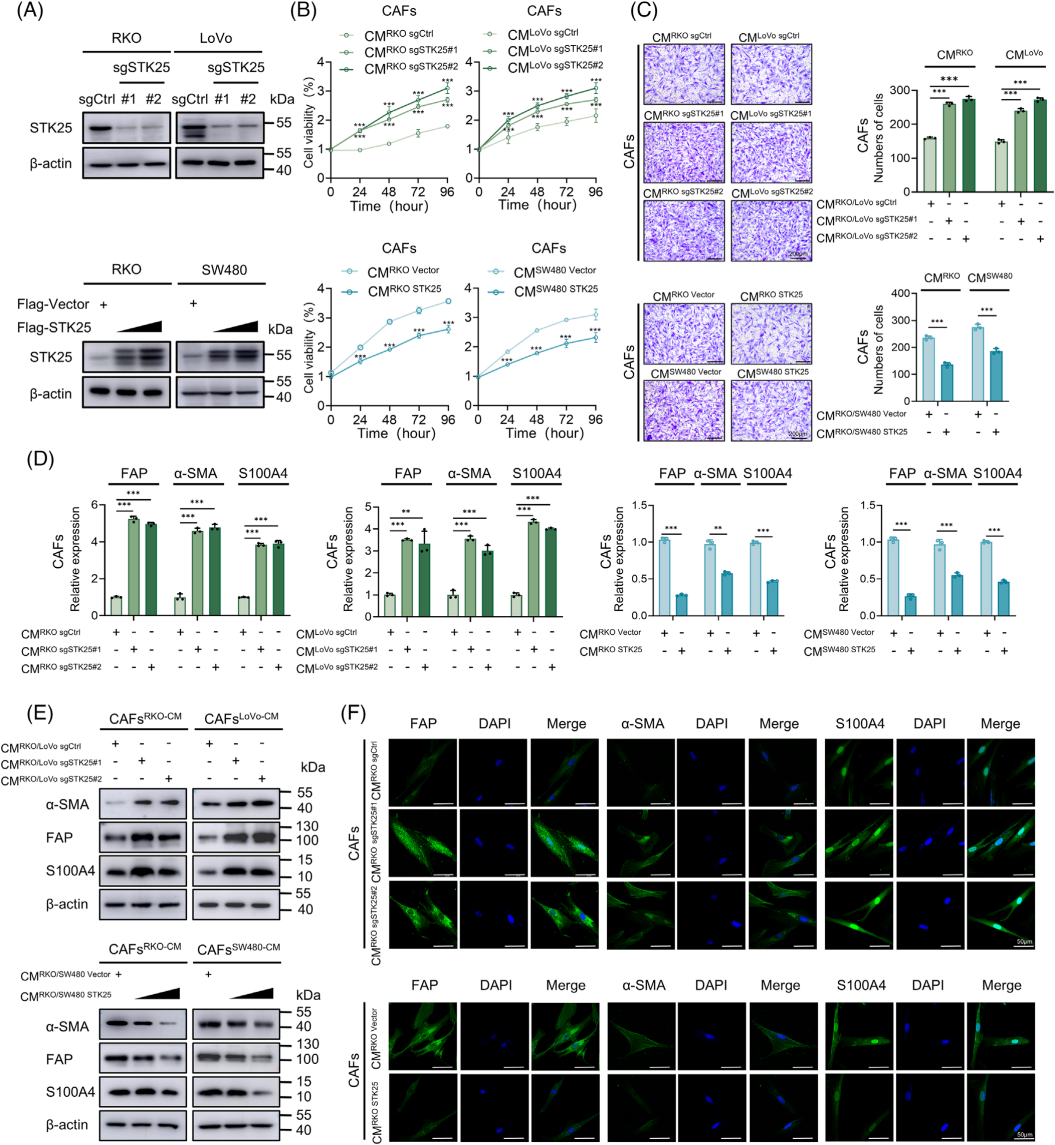
STK25 deficiency facilitated CAF proliferation, migration and activation. (A) Western blot analysis showing CRISPR/Cas9‐mediated STK25 knockout in RKO and LoVo cells, as well as STK25 overexpression in RKO and SW480 cells. (B and C) CCK‐8 (B) and transwell (C) assays used to evaluate CAF proliferation and migration treated with CM from STK25 knockdown or overexpressing colorectal cancer (CRC) cells. Graphs representing the quantification of migrated cells. Scale bar, 200 µm. (D and E) qRT‐PCR (D) and western blot (E) analysis of CAF activation markers, including α‐SMA, FAP and S100A4, following exposure to CM from the indicated CRC cells. (F) Representative immunofluorescence images of α‐SMA, FAP and S100A4 in CAFs cultured with CM from indicated RKO cells. Scale bar, 50 µm. Data are presented as the mean ± SD of at least three independent experiments. One‐way ANOVA and two‐way ANOVA were used for statistical analyses. **p < .01, ***p < .001.

To further investigate whether STK25 participates in CRC cell‐induced CAF activation, an indirect co‐culture system using CM was established. The efficiency of STK25 overexpression and knockdown in CRC cells was confirmed by western blot in CRC cells (Figures 1A and S1F). The results of CCK8 and transwell assays demonstrated STK25 depletion in CRC cells markedly enhanced CAFs proliferation and migration, whereas STK25 overexpression in CRC cells significantly suppressed these effects (Figure 1B,C). Moreover, qRT‐PCR and western blot results showed that CM from STK25‐knockdown CRC cells increased the mRNA and protein levels of FAP, α‐SMA and S100A4 in CAFs. In contrast, CM from STK25‐overexpressing CRC cells exhibited the opposite effects (Figures 1D,E and S1G). Consistent results were further validated by immunofluorescence analysis (Figures 1F and S1H). Collectively, these findings indicated that STK25 silencing in CRC cells facilitated CAF activation.

### 3.2 STK25 knockdown‐mediated CAF activation enhanced the proliferation and metastasis of CRC cells

**FIGURE 2:**
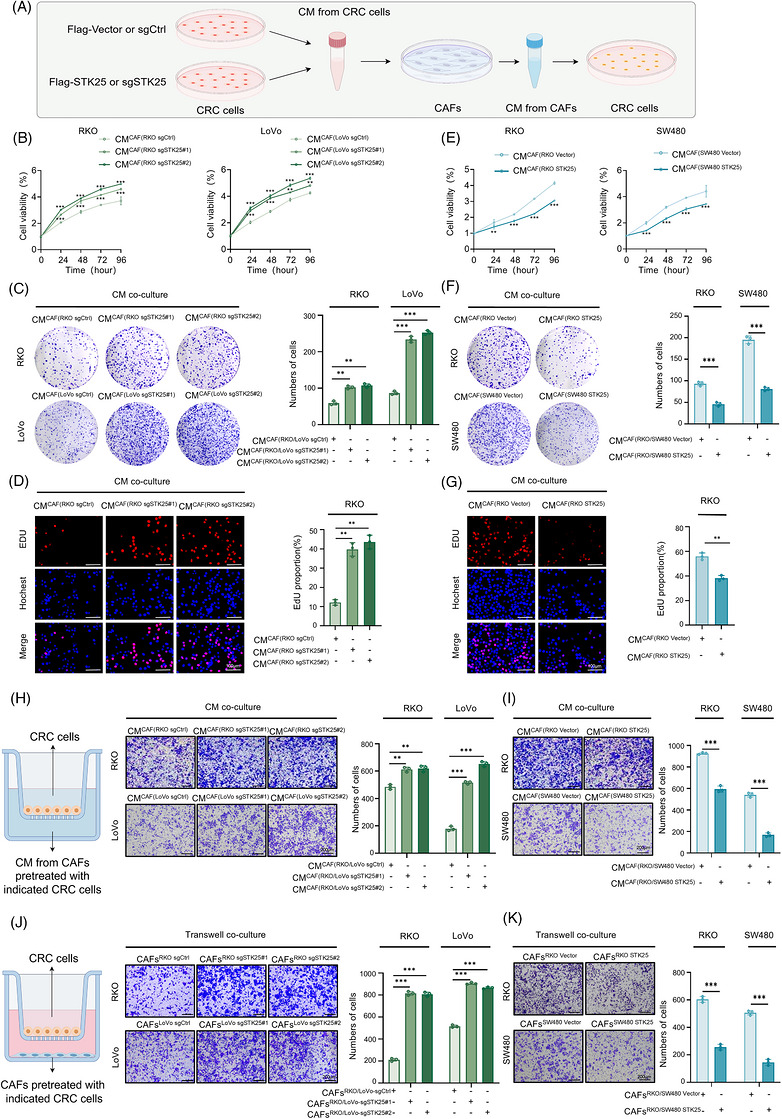
STK25 depletion‐mediated CAF activation promoted the proliferation and metastasis of CRC cells. (A) Schematic illustration of the co‐culture procedures for CRC cells and CAFs. (B–G) CCK‐8, colony formation, and EdU assays determined the proliferation of CRC cells co‐cultured with CM from CAFs stimulated by STK25 silencing (B–D) or STK25‐overexpressing CRC cells (E–G). Graphs showing quantification of colony numbers and the proportion of EdU‐positive cells. Scale bar, 100 µm. (H and I) Transwell assays evaluating migration of CRC cells exposed to CM from CAFs co‐cultured with STK25‐deficient or STK25‐overexpressing CRC cells. (J and K) Transwell migration assays of CRC cells co‐cultured with CAFs pretreated with indicated CRC cells. Graphs representing the quantification of migrated cells. Scale bar, 200 µm. Data are presented as the mean ± SD of at least three independent experiments. One‐way ANOVA and two‐way ANOVA were used for statistical analyses. **p < .01, ***p < .001.

Paracrine communication between CAFs and cancer cells facilitates tumour progression.^28^ To further investigate the reciprocal effects of activated CAFs on CRC cells, CRC cells were incubated in CM collected from CAFs that had been previously exposed to indicated tumour cells for 48 h (Figure 2A).

Notably, RKO and LoVo cells incubated with CM from CAFs co‐cultured with STK25‐knockdown CRC cells (CM‐CAF/CRC sgSTK25) exhibited a higher proliferative rate than those with CM from CAFs co‐cultured with control CRC cells (CM‐CAF/CRC sgCtrl) (Figure 2B–D). However, CRC cells cultured in CM from CAFs co‐cultured with STK25‐overexpressing CRC cells (CM‐CAF/CRC STK25) showed reduced proliferation phenotypes (Figure 2E–G). Subsequently, in transwell assays, CM obtained from CAFs co‐cultured with indicated CRC cells was added to the lower chamber, and CRC cells were seeded in the upper chamber. The results demonstrated that the number of migratory CRC cells was significantly increased when cells were stimulated with CM‐CAF/CRC sgSTK25 (Figure 2H). In contrast, CRC cells stimulated with CM‐CAF/CRC STK25 exhibited the opposite trends (Figure 2I). Moreover, the transwell co‐culture system with CRC cells and CAFs, respectively, in the upper and bottom chambers showed similar results. It was shown that CAFs activated by STK25‐silencing tumour cells significantly increased the migration of CRC cells, while CAFs stimulated by STK25‐overexpressing tumour cells showed the opposite effects (Figure 2J,K).

Taken together, these results demonstrated that CAF activation induced by STK25‐knockdown CRC cells facilitated CRC proliferation and migration, indicating STK25 in CRC cells modulated tumour progression by mediating the crosstalk between tumour cells and CAFs.

### 3.3 STK25 down‐regulation enhanced CAF‐mediated CRC growth in vivo

**FIGURE 3:**
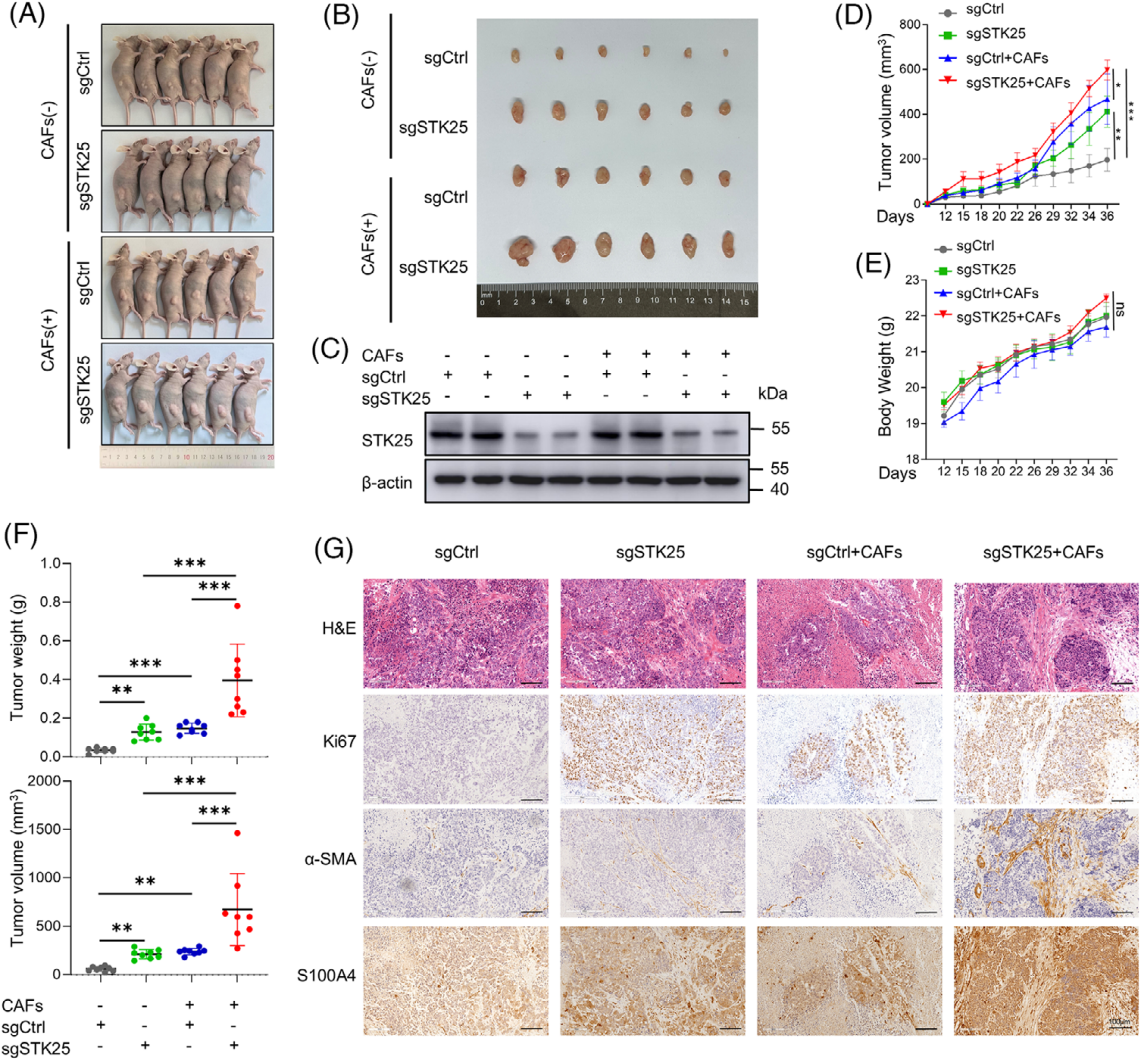
Knockout of STK25 in CRC cells enhanced CAF‐mediated CRC growth in vivo. (A and B) Nude mice were subcutaneously injected with sgSTK25 or sgCtrl–RKO cells mixed with or without CAFs (*n* = 8 per group). Representative images of mass morphology (A) and tumour tissues isolated from mice (B) in different treatment groups were displayed. (C) Western blot analysis of STK25 expression in the tumour tissues from mice. (D and E) Tumour volume and body weight were measured every 2–3 days and the tumour growth curves were plotted over time. (F) Tumour weight and volume of mice were recorded when mice were sacrificed at day 36. (G) Representative haematoxylin and eosin (H&E) images and immunohistochemical (IHC) staining of Ki67, α‐SMA and S100A4 in tumour tissues of mice from different groups. Graphs representing the quantification of IHC staining. Scale bars, 100 µm. One‐way ANOVA and two‐way ANOVA were used for statistical analyses. *p < .05, **p < .01, ***p < .001; ns, not significant.

Next, we investigated the role of STK25 in orchestrating CAF‐mediated CRC progression in vivo. sgSTK25–RKO cells or control cells mixed with or without CAFs were subcutaneously injected into nude mice. After 36 days, mice were sacrificed, and the subcutaneous tumour tissues were collected (Figure 3A,B). We confirmed that STK25 expression was markedly decreased in tumour tissues from mice subcutaneously injected with sgSTK25–RKO cells (Figure 3C). Tumour volume and body weight of mice in different groups were measured every 2–3 days. The tumour growth and body weight curves were plotted over time (Figure 3D,E). Our results showed that STK25 knockdown significantly increased tumour weight and volume compared with the control group. Moreover, STK25‐deficient RKO cells co‐injected with CAFs exhibited the most pronounced tumour‐promoting effects, without significant changes in body weight (Figure 3A–F). Subsequently, we assessed the correlation between STK25 expression and CAF activation in tumour tissues by IHC analysis. STK25 expression was negatively correlated with Ki67, α‐SMA, and S100A4 levels in tumour tissues. Notably, tumours generated by CAFs co‐injected with STK25‐knockdown CRC cells exhibited the highest staining intensity of Ki67, α‐SMA and S100A4 (Figures 3G and S2A–C). In general, these results indicated a synergistic effect of CAFs and STK25‐knockdown cancer cells on facilitating CRC progression in vivo.

### 3.4 Knockdown of STK25 in CRC cells promoted CAF activation via the AREG/EGFR axis

To further explore the underlying mechanisms by which STK25 regulates the crosstalk between CRC cells and CAFs, we analysed our previously published single‐cell RNA sequencing (scRNA‐seq) data of AOM/DSS‐induced colorectal tumours from wild‐type (WT) and STK25 global knockout (STK25^−/−^) mice, which are available from the NCBI GEO database (GSE277814).

**FIGURE 4:**
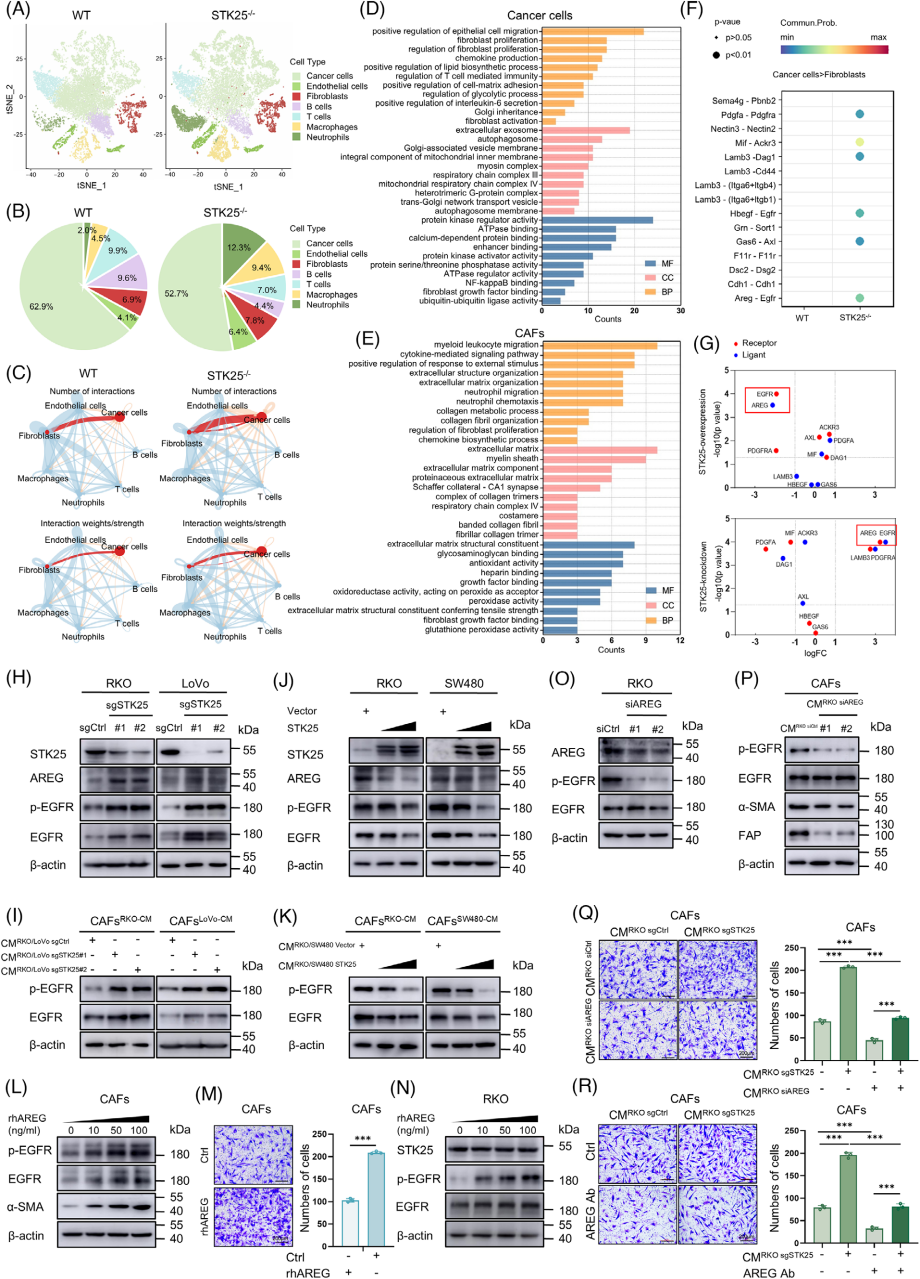
STK25‐mediated communication between tumour cells and CAFs depended on the AREG/EGFR axis. (A) tSNE plot of cell subsets acquired from the single‐cell RNA sequencing (scRNA‐seq) data of CRC tumours in WT and STK25^−/−^ mice treated with AOM/DSS (GEO accession number GSE277814). (B) The proportions of each cell type in AOM/DSS‐induced tumours of WT and STK25^−/−^ mice. (C) Cell–cell communication analysis among different cell types was performed using CellChat. The communication number and strength of interactions were visualised. (D and E) Gene Ontology (GO) enrichment analysis of differentially expression genes (DEGs) in cancer cells (D) and CAFs (E) from the scRNA‐seq dataset. (F) CellChat analysis used to predict enriched ligand–receptor pairs between cancer cells and CAFs. (G) mRNA levels of ligand and receptor genes were detected by qRT‐PCR in STK25‐overexpressing or STK25‐knockdown CRC cells and co‐cultured CAFs. A volcano plot illustrating fold changes and *p* values of the ligand and receptor genes. (H and I) Western blot analysis of the expression of STK25, AREG, p‐EGFR and EGFR in STK25‐knockdown CRC cells (H), as well as p‐EGFR and EGFR in co‐cultured CAFs (I). (J and K) The expression of indicated proteins in STK25‐overexpressing CRC cells transfected with 2 or 4 µg Flag‐STK25 plasmid (J) and co‐cultured CAFs (K). (L) The expression of p‐EGFR, EGFR and α‐SMA in CAFs treated with recombinant human AREG (rhAREG). (M) Transwell migration assays of CAFs under the treatment of rhAREG. Scale bars, 200 µm. (N) Western blot analysis of p‐EGFR and STK25 expression in RKO cells co‐cultured with CAF pretreated with rhAREG. (O and P) The levels of indicated proteins in AREG‐depleted RKO cells (O) and co‐cultured CAFs (P). (Q and R) Transwell assays to detect the migration of CAFs co‐cultured with CM from sgSTK25 CRC cells transfected with siAREG (Q) or treated with AREG antibody (R). Graphs representing the quantification of migrated cells. Scale bars, 200 µm. Data are presented as the mean ± SD of at least three independent experiments. One‐way ANOVA were used for statistical analysis. ***p < .001.

Unsupervised t‐distributed stochastic neighbour embedding (tSNE) analysis identified seven cell clusters in CRC tissues according to the specific cell markers, including epithelial cells (cancer cells), endothelial cells, fibroblasts, B cells, T cells, macrophages and neutrophils. Compared with WT mice, we observed a slight increase in the proportion of CAFs in STK25^−/−^ mice tumours (Figure 4A, B). Further subcluster analysis of CAFs revealed that STK25 knockout led to an increased proportion of the antigen‐presenting CAFs and vascular CAFs subpopulations, accompanied by a decreased proportion of myofibroblastic CAFs (myCAFs) and inflammatory CAFs (Figure S3A,B). To explore the differential interactions between tumour cells and CAFs in WT and STK25^−/−^ mice, we applied CellChat to visualise the communication number and strength of interactions. The analysis revealed that STK25 deletion substantially increased the number of interactions, but without significantly altering the overall communication strength between tumour cells and CAFs (Figure 4C).

Next, functional enrichment analysis was performed on the differentially expressed genes (DEGs). Gene Ontology (GO) enrichment analysis of tumour cells revealed that the DEGs were significantly enriched in biological processes related to fibroblast proliferation, regulation of fibroblast proliferation and fibroblast activation (Figure 4D). Similarly, GO enrichment analysis of CAFs indicated that the DEGs were associated with pathways involving extracellular structure organisation, ECM organisation, regulation of fibroblast proliferation, ECM and ECM structural constituents (Figure 4E). Collectively, these results suggested that STK25 depletion in tumour cells may modulate CAF activity in the TME, leading to tumour progression.

Furthermore, to identify potential cellular interactions between tumour cells and other cells induced by STK25 depletion, we performed an analysis according to receptor–ligand interactions using the scRNA‐seq data. The results showed that the enriched ligand–receptor pairs predicted by CellChat were significantly increased in the STK25^−/−^ group compared with the STK25 WT group, especially between the cancer cells and endothelial cells, as well as fibroblasts (Figure S3C). PDGFA–PDGFRA, MIF–ACKR3, LAMB3–DAG1, HBEGF–EGFR, GAS6–AXL and AREG–EGFR were enriched ligand–receptor pairs between cancer cells and fibroblasts (Figure 4F), which were also involved in “regulation of fibroblast proliferation” identified by GO enrichment analysis in these two cell populations (Figure 4D,E). Among them, AREG and EGFR exhibited the most significant changes, as validated by qRT‐PCR analysis in CRC cells and CAFs, respectively (Figures 4G and S3D).

Then, we performed in vitro experiments to further examine whether STK25‐mediated communication between tumour cells and CAFs depends on the AREG/EGFR axis. The results showed that STK25 knockdown significantly increased AREG and p‐EGFR expression in tumour cells, as well as elevated p‐EGFR levels in co‐cultured CAFs. In contrast, overexpression of STK25 in tumour cells exhibited the opposite effects (Figures 4H–K and S3E–H). qRT‐PCR analysis revealed consistent changes in AREG and EGFR mRNA levels in cancer cells and CAFs, respectively (Figure S3I). Moreover, as expected, the recombinant human AREG (rhAREG) significantly enhanced p‐EGFR levels and CAF markers in a dose‐dependent manner (Figures 4L and S3J), and promoted CAF migratory capacity (Figure 4M). Interestingly, CAFs pretreated with rhAREG promoted p‐EGFR expression in CRC cells without significant change in STK25 levels (Figure 4N). However, knockdown of AREG expression in tumour cells led to the decrease of p‐EGFR in both tumour cells and CM co‐cultured CAFs, in parallel with a reduction in CAF activation markers (Figures 4O,P and S3K,L). More importantly, AREG silencing in CRC cells or AREG antibody treatment reversed the STK25 depletion‐induced increase of CAF migration (Figure 4Q,R). Taken together, these findings indicated that STK25 restricts CAF activation depending on the AREG/EGFR axis between tumour cells and CAFs.

### 3.5 STK25 loss up‐regulated AREG expression via the NF‐κB signal pathway

According to the scRNA‐seq data from colorectal tumours in STK25^−/−^ and WT mice described above, GO analysis suggested a potential role of STK25 in NF‐κB‐related transcriptional processes (Figure 4D). The primary NF‐κB protein complexes responsible for regulating target gene transcription are NFκB1(p50)/RelA(p65) heterodimers, as well as p50 and p65 homodimers. Additionally, the NF‐κB signalling pathway has been reported to modulate CAF activation and contribute to the acquisition of malignant phenotypes.^29^, ^30^ Thus, we sought to determine the regulatory effect of STK25 on the NF‐κB signalling pathway in CRC.

**FIGURE 5:**
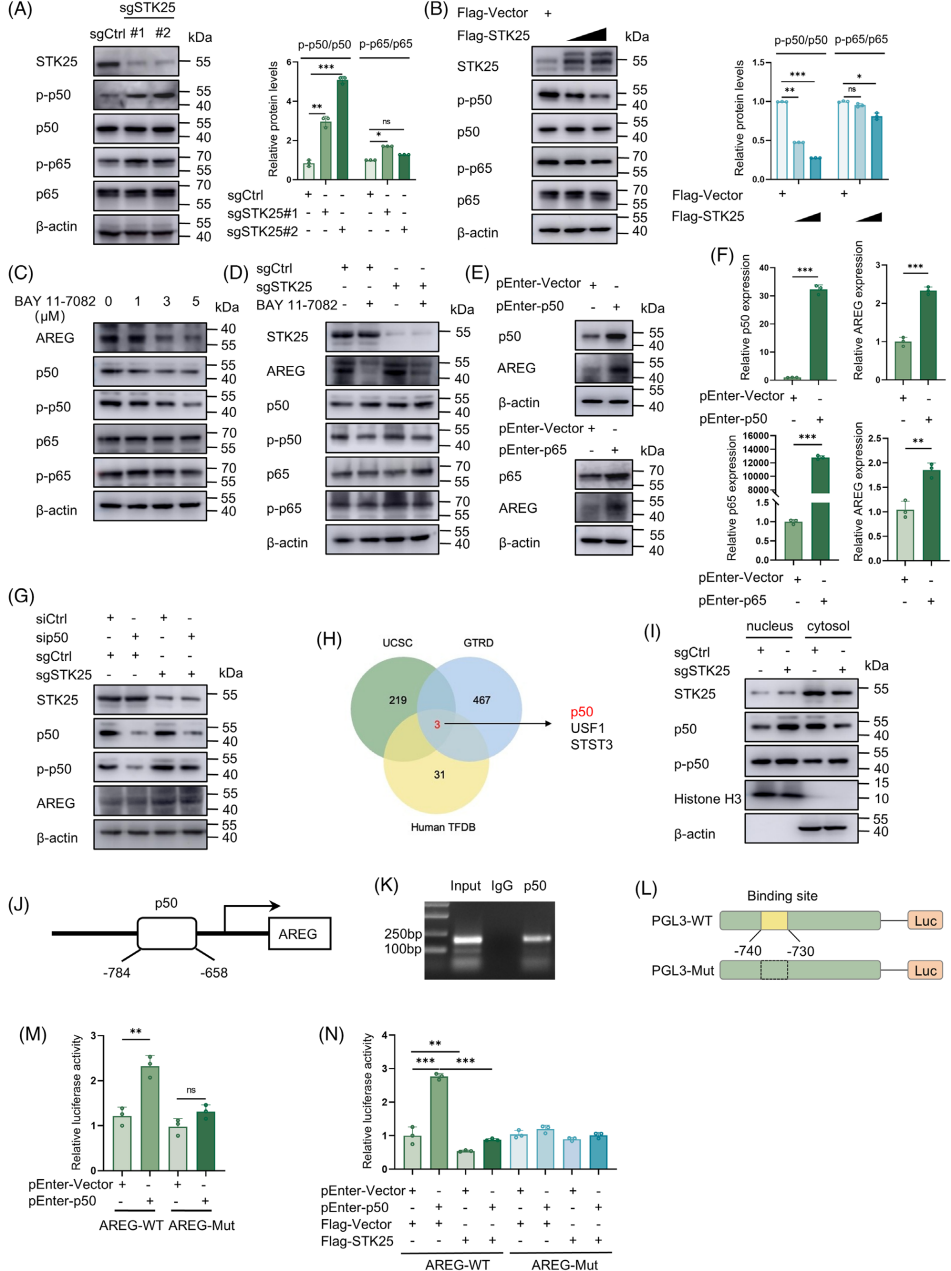
STK25 loss up‐regulated AREG expression via the NF‐κB signalling pathway. (A and B) Western blot analysis of p50, p65, phosphorylated p50 (p‐p50) and p65 (p‐p65) levels in STK25‐knockdown (A) and STK25‐overexpressing (B) CRC cells. (C and D) The expression of indicated proteins in CRC cells treated with BAY 11–7082 (C) or sgSTK25 CRC cells incubated with 5 µM BAY 11–7082 (D). (E and F) Western blot (E) and qRT‐PCR (F) analysis of protein and mRNA expression levels of AREG in CRC cells overexpressing p50 or p65. (G) AREG expression in sgSTK25 CRC cells transfected with sip50. (H) Venn diagram showed overlapping transcription factors predicted to regulate AREG expression from the UCSC, GTRD and Human TFDB databases. (I) p50 expression in nuclear and cytoplasmic fractions in sgCtrl and sgSTK25 CRC cells. (J) The predicted binding site for p50 in the AREG promoter. (K) Chromatin immunoprecipitation (ChIP) assay of p50 binding site at the AREG promoter in RKO cells. (L) Schematic illustration of wild‐type (PGL3‐WT) and mutated (PGL3‐Mut) AREG promoter luciferase reporter constructs. (M and N) Dual‐luciferase reporter assays indicated the effects of p50 overexpression on wild‐type (AREG‐WT) and mutated (AREG‐Mut, −740 to −730) promoter activity (M), and the role of STK25 in modulating this process (N). Data are presented as the mean ± SD of at least three independent experiments. Two‐tailed Student's *t*‐test and one‐way ANOVA were used for statistical analysis. *p < .05, **p < .01, ***p < .001; ns, not significant.

Western blot analysis revealed that STK25 knockdown enhanced the phosphorylation of p50 and p65, and STK25 overexpression produced the opposite effects in CRC cells, with p50 phosphorylation showing a more pronounced effect (Figure 5A,B). As expected, the NF‐κB inhibitor, BAY 11–7082, exerted a cytotoxic effect on CRC cells and inhibited the phosphorylation of p50 and p65 (Figures 5C and S4A). The addition of BAY 11–7082 could reverse the STK25 depletion‐induced increase of p‐p50, p‐p65 and AREG levels, as well as the enhanced CAF migration (Figures 5D and S4B). These data indicated the regulation of AREG by STK25 depends on the NF‐κB signalling pathway. Moreover, the overexpression of p50 and p65 significantly up‐regulated AREG expression at both the protein and mRNA levels, with p50 overexpression exhibiting a more pronounced effect on AREG expression at the mRNA level (Figures 5E,F and S4C). Thus, we investigated whether STK25 modulates AREG expression via p50. The depletion of STK25 could not promote AREG expression when knocking down p50 (Figures 5G and S4D), suggesting the crucial role of p50 in the regulation of STK25 on AREG expression. Additionally, we predicted the potential transcription factors regulating AREG expression using the UCSC, GTRD and Human TFDB databases. The Venn diagram illustrated the overlap of predicted transcription factors regulating AREG expression, including NF‐κB1 (p50), USF1 and STAT3 (Figure 5H). Furthermore, knockdown of STK25 enhanced the nuclear translocation of p50 (Figure 5I). Collectively, these findings suggested that p50 might function as a transcription factor to regulate AREG expression.

To further elucidate the role of p50 in AREG transcription, a putative p50 binding site within the AREG promoter was predicted using the JASPAR database (http://jaspar.genereg.net/) (Figure 5J). Next, chromatin immunoprecipitation (ChIP) assays revealed that p50 specifically binds to the AREG promoter region (−784 to −658 bp) (Figure 5K). However, no specific binding of p65 to this AREG promoter region was observed (Figure S4E). To precisely map the binding site, a mutant AREG promoter plasmid (AREG‐Mut, −740 to −730 bp) was generated for dual‐luciferase reporter assays (Figure 5L). As expected, overexpression of p50 enhanced the luciferase activity of the wild‐type AREG promoter (AREG‐WT), whereas the mutant promoter (AREG‐Mut) showed no significant promoter activity (Figure 5M). Furthermore, overexpression of STK25 attenuated p50‐mediated transcriptional activity at the AREG‐WT promoter but did not affect the AREG‐Mut promoter (Figure 5N). Taken together, these findings demonstrated that p50 enhances the transcriptional activity of the AREG promoter through direct binding to a specific promoter region, whereas STK25 suppresses this effect.

### 3.6 STK25 overexpression or AREG antibody treatment overcame CAF‐mediated cetuximab resistance in CRC

Cetuximab, a monoclonal antibody targeting EGFR, is widely used for the treatment of RAS wild‐type mCRC.^31^ Given the inhibitory role of STK25 in regulating the AREG/EGFR axis between tumour cells and CAFs, we next examined whether STK25 overexpression or treatment with a neutralising AREG antibody could enhance the anti‐tumour efficacy of cetuximab in CRC cells co‐cultured with CAFs.

**FIGURE 6:**
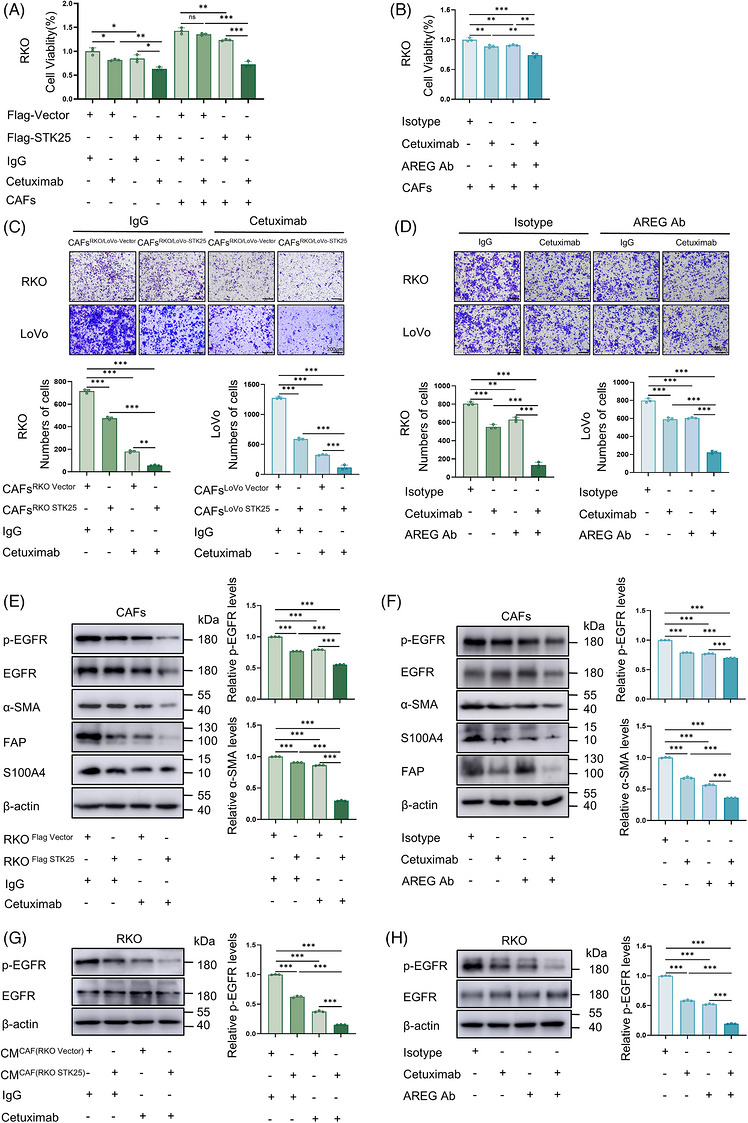
STK25 overexpression or AREG antibody treatment overcame CAF‐mediated cetuximab resistance in CRC. (A) CCK‐8 assays used to evaluate the proliferation of STK25‐overexpressing RKO cells cultured alone or co‐cultured with CAFs in the presence of 30 µg cetuximab. (B) CCK‐8 assays performed to examine the proliferative capacity of RKO cells co‐cultured with CAFs under the combined treatment of AREG antibody and 30 µg cetuximab. (C) Transwell assays to determine the migration of STK25‐overexpressing CRC cells co‐cultured with CAFs under the treatment of 30 µg cetuximab. (D) The migration of CRC cells co‐cultured with CAFs in the presence of AREG antibody and 30 µg cetuximab. Graphs representing the quantification of migrated cells. Scale bars, 200 µm. (E–H) Western blot analysis of the levels of the indicated proteins in CAFs (E and F) and the co‐cultured CRC cells transfected with 4 µg Flag‐STK25 plasmid (G) or treated with AREG antibody (H) upon cetuximab exposure. Data are presented as the mean ± SD of at least three independent experiments. One‐way ANOVA was used for statistical analyses. *p < .05, **p < .01, ***p < .001; ns, not significant.

The cell viability of RKO cells exposed to cetuximab was assessed using the CCK‐8 assay, with a nonspecific IgG antibody serving as a control. The IC50 of cetuximab for RKO cells was 151.1 µg/mL (Figure S5A), whereas nonspecific IgG exhibited no significant inhibitory effect on cell proliferation. The results showed that RKO cells treated with 30 µg/mL cetuximab or STK25 overexpression resulted in a reduction in cell growth by 18 and 15%, respectively. However, when co‐cultured with CAFs, tumour cells displayed decreased sensitivity to cetuximab. Importantly, overexpression of STK25 in tumour cells partially rescued the sensitivity of cetuximab (Figures 6A and S5B). Similarly, although cetuximab or treatment with a neutralising AREG antibody alone produced limited anti‐proliferative effects on CRC cells co‐cultured with CAFs, the combination of cetuximab and AREG antibody significantly enhanced cetuximab‐induced cytotoxicity in tumour cells (Figure 6B).

Subsequently, transwell migration assays demonstrated that cetuximab treatment or STK25 overexpression inhibited the migration of CRC cells that were co‐cultured with CAFs. STK25 overexpression combined with cetuximab treatment exerted the most pronounced inhibitory effect on tumour cell migration, indicating enhanced sensitivity to cetuximab (Figure 6C). Similarly, treatment with a neutralising AREG antibody facilitated the inhibitory effect of cetuximab on CRC cell migration when tumour cells were co‐cultured with CAFs (Figure 6D). Moreover, in vivo experiments demonstrated that tumours derived from CRC cells and CAFs with a relatively low level of STK25 exhibited significant resistance to cetuximab (Figure S5C–F).

We next sought to determine the regulatory effect of STK25 expression or cetuximab treatment on EGFR phosphorylation and CAF activation. In the co‐culture system of CRC cells and CAFs, STK25 overexpression or cetuximab exposure partially inhibited EGFR phosphorylation and the expression of α‐SMA, FAP and S100A4 in CAFs. Moreover, the combination of STK25 overexpression and cetuximab treatment showed the most inhibiting effects on EGFR phosphorylation and CAF activation (Figures 6E and S5G). Consistently, under the treatment of AREG‐neutralising antibody and cetuximab, similar trends were identified in the levels of phosphorylated EGFR and markers of CAF activation (Figures 6F and S5H). Additionally, STK25 overexpression or AREG‐neutralising antibody treatment facilitated the inhibitory effects of cetuximab on EGFR phosphorylation in RKO cells, which were co‐cultured with CAFs (Figure 6G,H).

These results suggested that STK25 overexpression or AREG‐neutralising antibody treatment apparently enhanced the anti‐tumour efficacy of cetuximab on tumour cells in the presence of CAFs.

### 3.7 STK25 overexpression enhanced the therapy response to cetuximab in patients with CRC

**FIGURE 7:**
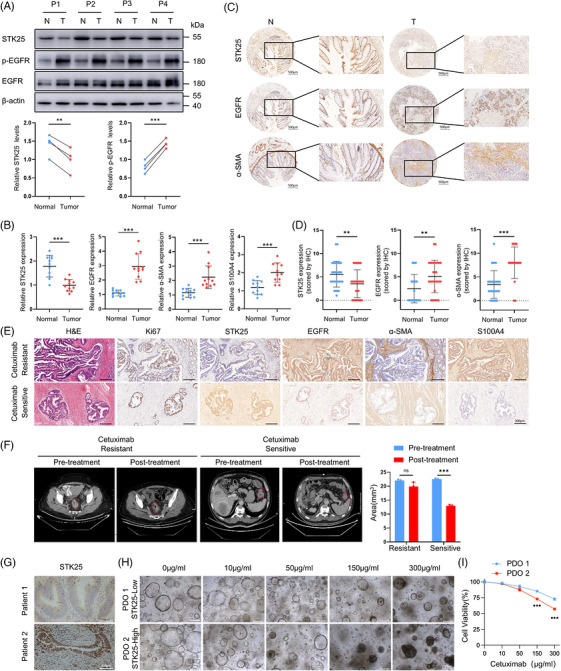
STK25 overexpression enhanced the therapeutic response to cetuximab in patients with CRC. (A) The protein levels of STK25, EGFR and p‐EGFR protein levels in four paired tumour tissues and corresponding adjacent normal tissues from CRC patients. Graphs representing the quantification of the intensity of protein bands. (B) The mRNA levels of STK25, EGFR, α‐SMA and S100A4 in 11 paired tumour tissues and matched adjacent normal tissues. (C and D) Representative IHC staining images (C) and quantification (D) of STK25, α‐SMA and EGFR staining in tumour tissues and adjacent normal tissues from CRC patients. Scale bar, 500 µm. (E) Representative H&E images and IHC staining of Ki67, STK25, EGFR, α‐SMA and S100A4 in CRC tissues from cetuximab‐resistant and cetuximab‐sensitive patients. (F) Representative computed tomography (CT) images of CRC patients from (E) before and after cetuximab therapy. Graphs representing the quantification of tumour volume. Scale bars, 300 µm. (G–I) Representative images of IHC for STK25 in CRC tissues (G), and the organoids derived from the corresponding patients were treated with cetuximab in vitro (H). Cell viability was evaluated by measuring ATP levels (I). Scale bars, 300 µm (G), 200 µm (H). Data are presented as the mean ± SD of at least three independent experiments. Two‐tailed Student's *t*‐test and two‐way ANOVA were used for statistical analyses. **p < .01, ***p < .001.

To assess the clinical relevance of STK25 in CRC patients, western blot analysis was performed on four paired CRC and adjacent normal tissues. The results revealed that tumours with low STK25 expression exhibited elevated levels of EGFR and p‐EGFR (Figure 7A). Consistently, qRT‐PCR analysis of 11 paired CRC tissue samples further revealed comparatively lower STK25 expression and higher expression of EGFR, α‐SMA and S100A4 in 11 CRC tissue samples compared with those in paired normal tissues (Figure 7B).

Moreover, IHC staining was performed on CRC tissue arrays using antibodies against STK25, α‐SMA and EGFR. The results showed that STK25 expression was markedly lower in CRC tissues compared with adjacent normal tissues, whereas α‐SMA and EGFR levels were significantly higher in tumour tissues (Figure 7C,D).

Subsequently, we determined the relationship between the STK25/AREG/EGFR axis and the therapeutic efficacy of cetuximab in CRC patients. We examined CRC patients undergoing cetuximab treatment and measured the levels of Ki67, STK25, EGFR, α‐SMA and S100A4 by IHC in CRC tissues (Figures 7E and S6A). Postoperative pathological assessment revealed that patients exhibiting high STK25 expression with low EGFR, α‐SMA and S100A4 levels showed a favourable response to cetuximab therapy. Consistently, computed tomography (CT) imaging confirmed these findings, demonstrating reduced tumour volume in patients with high STK25 expression following cetuximab treatment (Figure 7F). Moreover, patient‐derived organoids co‐cultured with CAFs confirmed the association between high STK25 level and superior cetuximab responses (Figure 7G–I). Additionally, the analysis of GSE108277 revealed that mCRC patient‐derived xenografts with high STK25 have better responses to cetuximab treatment than those with low STK25, as shown in the partial response subgroup compared with the stable disease subgroup (Figure S6B). Similarly, the analysis of GSE262796 datasets suggested that STK25 expression was higher in cetuximab‐sensitive LIM1215 cells than that in cetuximab‐resistant LIM1215 cells (Figure S6C). Collectively, these results confirmed that CRC patients with high STK25 expression may potentially benefit from cetuximab treatment.

## 4 DISCUSSION

**FIGURE 8:**
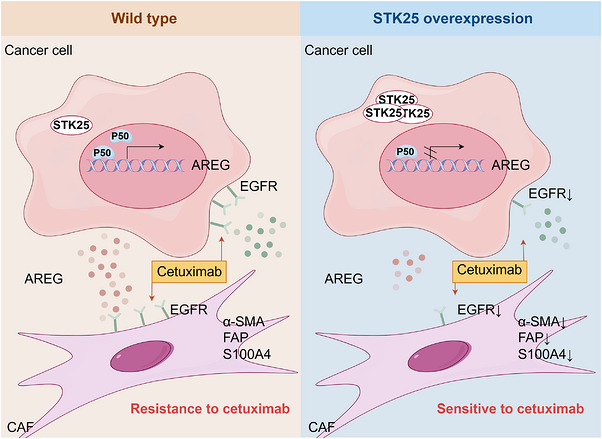
Schematic diagram depicted the mechanism by which STK25 overexpression inhibits CAF activation via the AREG/EGFR axis between tumour cells and CAFs. STK25 transcriptionally regulates AREG expression through NF‐κB (p50) activation, thereby enhancing the sensitivity of CRC to cetuximab therapy.

Tumour development involves phenotypic alterations and dynamic changes in cell–cell interactions within both the tumour cells and the stromal compartment. Our present study identified that overexpression of STK25 in CRC cells inhibited CAF activation via the AREG/EGFR axis, thereby suppressing tumour proliferation and migration. Mechanistically, STK25 overexpression suppressed the transcriptional activity of p50 at the AREG promoter. Moreover, either STK25 overexpression or AREG antibody treatment effectively overcame CAF‐mediated cetuximab resistance in CRC (Figure 8). Clinically, CRC tissues with high STK25 expression and low CAF activity might correlate with increased sensitivity to cetuximab therapy.

CAFs are critical cellular components within the TME and drive multiple pro‐tumourigenic processes, including tumour growth, angiogenesis, metastasis and drug resistance.^28^, ^32^, ^33^, ^34^ It has been well established that tumour progression is regulated by the interactions between tumour cells and CAFs, which are complex and heterogeneous in the TME across different cancer types. The CAFs in HCC are mainly derived from activated hepatic stellate cells and crosstalk with tumour cells via paracrine cytokines, exosomes and positive feedback loops. CAFs could enhance HCC cell stemness and the construction of an immunosuppressive microenvironment.^35^ In CRC, the major source of CAFs was derived from intestinal pericryptal Lepr‐lineage cells.^36^ The crosstalk between CAFs and CRC cells triggers tumour aggressiveness and therapy resistance through cytokines, exosomes and ECM remodelling.^37^ Due to the distinct origins of CAFs in HCC and CRC, this heterogeneity may partly account for the different functions of STK25 in these two tumour types.

In regard to the pathways between tumour cells and CAFs, Liu et al. found that ZNF37A‐mediated activation of TGF‐β signalling in CRC cells promoted stromal fibroblasts, thereby facilitating tumour metastasis.^38^ Similarly, silencing ZNF32 in breast cancer cells induced the transformation of NFs into CAFs by the modulation of TGFB1 transcription.^39^ Additionally, AREG has been identified as a ligand of EGFR to activate myCAFs, which promoted PDAC metastasis.^40^ The AREG/EGFR axis enabled Treg cells to induce a profibrotic and immunosuppressive phenotype upon CAFs, constituting a critical barrier for cancer immunotherapy.^41^ In this study, we demonstrated that STK25 depletion up‐regulated AREG expression in CRC cells, resulting in the activation of CAFs, and subsequently promoted tumour proliferation and migration, indicating that the STK25‐regulated AREG/EGFR axis mediated the communication between CRC cells and CAFs.

As a serine/threonine kinase, STK25 has multiple substrates, including PD‐L1, GOLPH3, YAP1 and MST1/2.^20^, ^22^, ^27^ The phosphorylation of these proteins was positively associated with STK25 levels. However, in this study, we found that STK25 suppressed the phosphorylation of both p65 and p50, which comprise the NF‐κB complex, thereby inhibiting AREG transcription. This observation suggests an indirect interaction between STK25 and p50. Thus, there might be other molecules involved in the regulation of STK25 to p50. Consistently, although the binding of Ser/Thr kinase ULK1 to raptor induced the phosphorylation of raptor, ULK1 inhibited the kinase activity of mTORC1, resulting in reduced phosphorylation of S6K1 and 4EBP1.^42^ Moreover, protein kinases can indirectly inhibit the phosphorylation of proteins by phosphorylating and activating downstream phosphatases. CaMKII phosphorylated and activated protein kinase phosphatase, CaMKPase, to facilitate the dephosphorylation activity towards CaMKII itself, forming a negative feedback loop.^43^ Therefore, further characterisation is required to determine the exact function of STK25 in the negative regulation of p50.

Increasing evidence indicates that there are associations between the efficacy of cetuximab treatment and tumour immunity. A previous study demonstrated that cetuximab, an IgG1 isotype monoclonal antibody, modulates immunity by triggering ADCC and priming innate and adaptive anti‐tumour immune responses.^44^ Additionally, cetuximab combined with chemotherapy stimulates an efficient antigen‐specific cytotoxic T‐lymphocyte response in colon cancer cells.^45^ Our previous study revealed that STK25 deficiency impairs CD8^+^ T cell‐induced anti‐tumour immunity to regulate the efficacy of ICB therapy.^27^ Therefore, the potential of STK25 in immune responses to modulate the effect of cetuximab needs further investigation.

In patients with the RAS/BRAF wild‐type mCRC, combining an anti‐EGFR monoclonal antibody (cetuximab or panitumumab) with chemotherapy represents a first‐line therapeutic strategy.^46^ Nevertheless, many patients who initially exhibit a positive response to cetuximab ultimately develop acquired resistance to the treatment.^47^ Activated CAFs have been implicated in driving resistance to the EGFR‐targeted therapies, including monoclonal antibodies (e.g., cetuximab) and tyrosine kinase inhibitors (e.g., gefitinib).^10^, ^11^, ^12^, ^13^, ^14^ Strategies combining targeted inhibitors with anti‐EGFR therapy have shown promise to overcome CAF‐mediated resistance to the EGFR‐targeted therapies. It was demonstrated that dual blockade of IGF‐1/IGF1R and HGF/c‐MET overcame CAF‐induced EMT and EGFR tyrosine kinase inhibition (EGFR‐TKI) resistance.^12^ IGF2BP2 inhibitor overcame CAF‐mediated cetuximab resistance in OSCC.^13^ MMP‐1 inhibitor significantly increased the sensitivity to cetuximab in the presence of CAFs in head and neck squamous cell carcinoma.^14^ Blocking the TGF‐β pathway with SIS3 enhanced cetuximab efficacy and suppressed tumour progression in preclinical models of head and neck cancer.^48^ Consistently, in this study, overexpression of STK25 in tumour cells significantly enhanced the anti‐tumour efficacy of cetuximab in the presence of CAFs, indicating the potential role of STK25 in overcoming cetuximab resistance in CRC.

As the ligand of EGFR, AREG is overexpressed in multiple tumour types, including gastrointestinal cancer, breast cancer, lung cancer and liver cancer. Previous studies showed that elevated AREG levels may predict resistance to gefitinib and correlate with poor prognosis in patients with NSCLC.^49^ In RAS wild‐type mCRC, AREG has been reported to reduce cellular sensitivity to cetuximab.^50^, ^51^ Inhibition of AREG activity was essential to overcome gefitinib resistance in NSCLC and HCC.^49^, ^52^ Notably, combining EGFR‐TKI with anti‐AREG therapy effectively suppressed radiotherapy‐induced metastatic growth in NSCLC.^53^ Moreover, cetuximab resistance mediated by Rablla‐exosomes in CRC cells could be partially reversed by the treatment of anti‐AREG antibody.^51^ In this study, anti‐AREG treatment significantly abolished the CAF‐induced cetuximab resistance in tumour cells co‐cultured with CAFs. Collectively, these findings highlighted the potential of targeting AREG to enhance the efficacy of EGFR inhibitors in patients with CRC.

Nevertheless, there are several limitations in our study. To validate whether STK25 can serve as a predictor for the efficacy of EGFR‐targeted therapy, studies involving larger cohorts of CRC patients treated with cetuximab are warranted. Moreover, further studies are required to clarify the therapeutic potential of cetuximab combined with STK25‐targeted therapies to treat CRC patients.

In summary, our study demonstrated that STK25 inhibited tumour progression and CAF activation via the AREG/EGFR axis, which was transcriptionally dependent on the NF‐κB activation. Moreover, overexpression of STK25 abolished CAF‐induced cetuximab resistance. Thus, STK25 may serve as a predictive indicator for the efficacy of EGFR‐targeted therapies in CRC patients, and the combination strategies targeting STK25 could potentially enhance the therapeutic efficacy of cetuximab in patients with CRC.

## AUTHOR CONTRIBUTIONS

Yifan Hou, Jiangbo Chen and Hao Hao performed the experiments, investigation, data curation and statistical analysis and wrote the original draft. Tongkun Song, Lin Song, Pu Xing and Kai Weng performed experiments, methodology and formal analysis. Yumeng Ran, Xinying Yang, Xiaowen Qiao, Ruibin Yao, Kai Xu and Jie Chen contributed to the methodology, software and statistical analysis. Hong Yang, Lei Chen and Jiabo Di provided resources. Beihai Jiang, Xiangqian Su, Kai Xu, Hong Yang and Jiangbo Chen participated in study design and funding acquisition. Beihai Jiang, Xiangqian Su and Kai Xu supervised the project and reviewed the manuscript. All authors read and approved the final manuscript.

## CONFLICT OF INTEREST STATEMENT

The authors declare no conflicts of interest.

## CONSENT FOR PUBLICATION

The authors have nothing to report.

## ETHICS STATEMENT

This study was approved and supervised by the Research Ethics Committee of Peking University Cancer Hospital & Institute, Beijing, China (2021KT136), and informed consent was obtained from all participants. The animal studies were approved by the Ethics Committee of the Peking University Cancer Hospital & Institute (2021KT134).

## Supporting information



Supporting Information
Additional supporting information can be found online in the Supporting Information section at the end of this article.

## Data Availability

All data generated or analysed during this study are included in this article and its Supporting Information.
