# Supplementary material for: STK25 inhibits cancer‐associated fibroblast activation to overcome cetuximab resistance in colorectal cancer
Source: Clin Transl Med. 2026 Apr 29;16(5):e70678. doi: 10.1002/ctm2.70678 (PMC13129231; doi:10.1002/ctm2.70678)
Supplement: Supplementary file 1 — Supporting Information Additional supporting information can be found online in the Supporting Information section at the end of this article. [file CTM2-16-e70678-s001.pdf]

Supplemental Appendix

STK25 inhibits cancer-associated fibroblast activation to overcome cetuximab resistance in colorectal cancer

Contents in Supplementary File

1、Supplementary Materials and Methods

2、Supplementary Figure S1-6 and Legends

3、Supplementary Table S1-6

Supplementary Materials and Methods

Materials and Methods

Cell lines and reagents

Human CRC cell lines (RKO, LoVo, and SW480) were obtained from the American Type Culture Collection (ATCC, Manassas, VA, USA). Cells were maintained in Dulbecco's Modified Eagle Medium (DMEM; HyClone, Logan, UT, USA) supplemented with 10% fetal bovine serum (FBS; Gibco, USA) and 1% penicillin-streptomycin (Gibco, USA). All cell cultures were maintained at 37 °C in a humidified incubator with 5% CO_2. The following reagents were used in this study: human amphiregulin antibody (MAB-262; R&D Systems, USA), recombinant amphiregulin protein (HY-P7002; MedChemExpress, China), BAY 11-7082 (S2913; Selleck, Shanghai, China), and cetuximab (Merck, Darmstadt, Germany).

Plasmid, small interfering RNA (siRNA), and lentivirus transfections

The full-length human STK25 cDNA was cloned into the pCMV-3Tag-1A vector to generate the STK25 overexpression plasmid. The p50 overexpression plasmid (CH803620) was obtained from WZ Biosciences Inc., and the p65 construct was generously provided by Prof. Jun Zhang (Department of Immunology, School of Basic Medical Sciences, Peking University). For STK25 depletion, two single guide RNAs (sgRNAs) were designed and synthesized by Shanghai Jikai Gene Chemical Technology Co., Ltd. Small interfering RNAs (siRNAs) specifically targeting AREG

and p50 were purchased from Ribobio Biologicals (Guangzhou, China). The sgRNA and siRNA sequences are listed in Table S5.

Transfections of plasmids and siRNAs were performed using EndoFectin™ Max (GeneCopoeia, Rockville, MD, USA) in accordance with the manufacturer's protocol. For CRISPR/Cas9-mediated STK25 knockout, cells were transduced with lentiviruses carrying sgRNAs for 72 h, followed by puromycin (4 μg/mL) selection for 7 days.

Conditioned medium (CM) preparation

CAFs or CRC cells were cultured to approximately 70% confluence and incubated overnight. The cells were then washed twice with PBS and replaced with fresh DMEM for an additional 48 h to collect CM. The supernatant was clarified by centrifugation at 3,000 × g for 10 min, aliquoted, and either used immediately or stored at −80 °C for subsequent experiments.

Cell proliferation assay

Cell proliferation was assessed using the Cell Counting Kit-8 (CCK-8), colony formation, and EdU staining assays. The detailed experimental procedures were described previously.[1] The concentration of cetuximab used in CRC cells was 1/5 of the IC50 value, a dosing regimen described by Alam et al.[2]

Cell migration assay

Wound healing assays and transwell migration assays were conducted to evaluate

cell migration capacity, the procedures have been previously described.[1, 3] In the transwell migration assay, cells were suspended in serum-free medium and plated in the upper chambers, while the lower chambers contained either CM as a chemoattractant or pretreated cells to provide chemotactic signals.

Quantitative real-time PCR (qRT-PCR)

Total RNA was extracted from CAFs and CRC cells using TRIzol reagent (Invitrogen) according to the manufacturer's instructions. Reverse transcription was performed using the Reverse Transcription Kit (Promega, Madison, WI, USA). qRT-PCR was performed as previously described.[1] GAPDH served as the internal reference gene. Primer sequences used in this study are listed in Table S6.

Western blot and immunofluorescence

The detailed procedures of western blot and immunofluorescence were described in a previous study.[1] Nuclear and cytoplasmic proteins were extracted using the Nuclear and Cytoplasmic Protein Extraction Kit (P0028; Beyotime, Shanghai, China) according to the manufacturer's protocol. The following primary antibodies were used for western blot: STK25 (1:1000; Cat#25821-1-AP, Proteintech), FAP (1:1000; Cat #84018, Proteintech), S100A4 (1:5000; Cat #16105, Proteintech), α-SMA (1:1000; Cat #19245T, CST), EGFR (1:5000; Cat #66455, Proteintech), p-EGFR (1:1000; Cat #3777, CST), AREG (1:3000; Cat #66433, Proteintech), p50 (1:1000; Cat #13586T, CST), p-p50 (1:1000; Cat #5512R, Bioss), p65 (1:1000; Cat #8242T, CST), p-p65 (1:1000; Cat

#3033T, CST), Histone H3 (1:2000; Cat #4499, CST). β-actin (1:5000; Cat #A1978, Sigma-Aldrich) was used as a control. Secondary antibodies included Goat anti-mouse IgG (1:4000; Cat #ZB-2305, ZSGB-BIO, China) or goat anti-rabbit IgG (1:4000; Cat #ZB-2301, ZSGB-BIO, China). The densities of protein bands were quantified using ImageJ software. For immunofluorescence assays, the following primary antibodies were employed: anti-FAP (1:200; Cat# 84018, Proteintech), anti-S100A4 (1:200; Cat# 16105, Proteintech), and anti-α-SMA (1:200; Cat# 19245T, CST). An anti-rabbit IgG secondary antibody (1:1000; Cat# 4412S, CST) was used for detection.

Immunohistochemistry (IHC)

Human CRC tissues and mouse subcutaneous tumors were fixed, paraffin-embedded, and sectioned at 4 μm thickness. Sections were then subjected to hematoxylin and eosin (H&E) staining or IHC following standard protocols. IHC was performed using primary antibodies against STK25 (1:1000, Santa Cruz, sc-271196), EGFR (1:5000, Proteintech, 66455), S100A4 (1:5000, Proteintech, 16105), and α-SMA (1:1000, CST, 19245T). The staining intensity was semi-quantitatively assessed by evaluating both the intensity (graded as 0: negative, 1: weak, 2: strong) and the proportion of positive cells (scored as 0: 0%, 1: ≤25%, 2: 26-50%, 3: 51-75%, 4: ≥76%). The final IHC score (range 0-8) was calculated by multiplying these two scores. The results analyzed by the two independent pathologists showed a high degree of consistency. All discrepancies were re-examined to reach a final consensus.

Chromatin immunoprecipitation (ChIP) assays

ChIP assay was conducted using the SimpleChIP® Plus Sonication Chromatin IP Kit (9002; CST, MA, USA). The anti-p50 antibody (Cat#13586, CST) and p65 (1:1000; Cat #8242T, CST) were used for ChIP. Briefly, approximately 2 × 10^7 RKO cells cultured in 10-cm^2 dishes were fixed with 1% formaldehyde for 10 min and washed with PBS. Cells were then harvested and lysed, and chromatin was digested with micrococcal nuclease, followed by sonication to generate soluble chromatin fragments. The lysates were subsequently incubated with the primary antibody overnight at 4°C for immunoprecipitation, and the resulting complexes were captured using agarose beads for 2h at 4°C. Crosslinks were reversed by elution at 65 °C for 30 min, and DNA fragments were purified using a spin column.

Luciferase reporter assay

Potential p50 binding sites within the 1,000 bp region upstream of the AREG transcription start site were predicted using the JASPAR database. The wild-type AREG promoter fragment (−1000 to 0 bp) and a mutant fragment containing a deletion of the predicted p50-binding site (−740 to −730 bp, GGGAGTCTCCT) were cloned into the pGL3-basic plasmid. Cells were seeded in 24-well plates and transfected with the luciferase reporter constructs. After 48 hours of transfection, cells were harvested, and luciferase activity was examined using the Dual-Luciferase Reporter Assay System (Promega, WI, USA).

Organoid cultures

CRC tissues for organoid establishment were collected from patients after surgical resection, and organoid culture was performed as previously described.[1] The organoids co-cultured with CAFs were treated with different concentrations of cetuximab and photographed to evaluate drug sensitivity. Organoid cell activity was assessed using the Cell Titer-Glo-3D Cell Viability Assay (Promega, Cat# G9683) in accordance with the manufacturer's protocol.

GEO database analysis

The scRNA-seq dataset (GSE277814) and Gene expression datasets (GSE108277 and GSE262796) were obtained from the NCBI Gene Expression Omnibus (GEO) database (https://www.ncbi.nlm.nih.gov/geo/). The scRNA-seq data was established in our previous study by Qiao et al.[4] Subsequent data analysis was performed using the R package "Seurat".[5] Gene expression matrices for GSE108277 and GSE262796 were downloaded from the GEO database, and probe IDs were subsequently converted to corresponding gene symbols. Differences in STK25 expression between different groups were compared using the two-tailed Student's t-test.

References

1. Xing P, Chen J, Hao H, et al. MTCH2 Deficiency Promotes E2F4/TFRC-Mediated Ferroptosis and Sensitizes Colorectal Cancer Liver Metastasis to Sorafenib. *Adv Sci (Weinh)*. 2025:e00019.
2. Alam KJ, Mo JS, Han SH, et al. MicroRNA 375 regulates proliferation and migration of colon cancer cells by suppressing the CTGF-EGFR signaling pathway. *Int J Cancer.* 2017;141(8):1614-1629.
3. Peng L, Zhao M, Liu T, et al. A stop-gain mutation in GXYLT1 promotes metastasis of colorectal cancer via the MAPK pathway. *Cell Death Dis.* 2022;13(4):395.
4. Qiao X, Xing P, Hao H, et al. STK25 Loss Augments Anti-PD-1 Therapy Efficacy by Regulating PD-L1 Stability in Colorectal Cancer. *Adv Sci (Weinh).* 2025:e03891.
5. Wang L, Chen B, He J, et al. ZFP36L1 promotes non-small cell lung cancer progression under hypoxia by modulating CXCL9:SPP1 polarity: A single-cell transcriptomic study. *Clin Transl Med.* 2026;16(4):e70642.

Supplementary Figures:

Figure S1 related to Figure 1

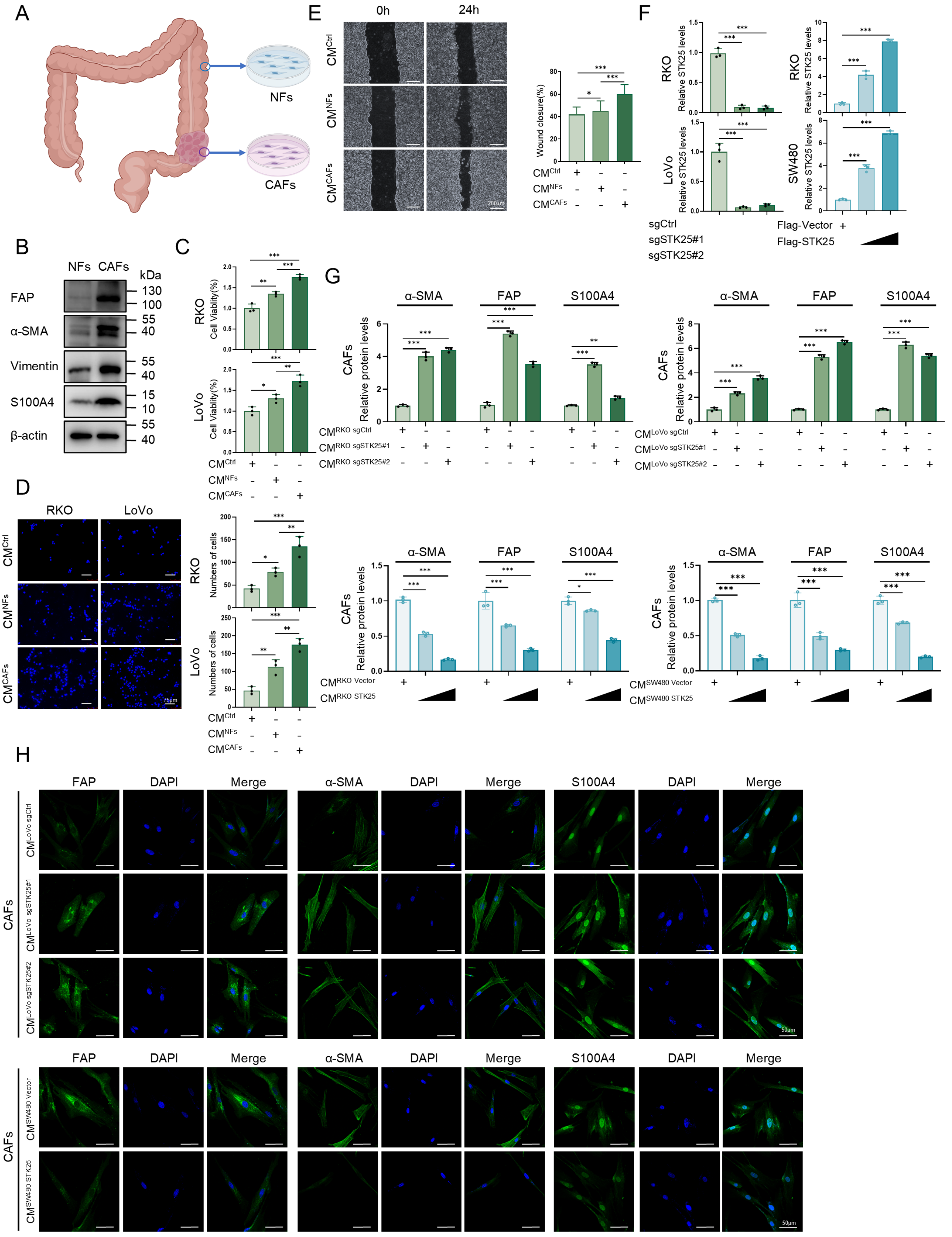

Figure S1 related to Figure 1. STK25 knockdown in CRC cells activated CAFs isolated from CRC tissues. (A) Schematic diagram illustrated the isolation of fibroblasts from CRC tissues and paired adjacent normal tissues, defined as CAFs and normal fibroblasts (NFs), respectively. (B) Western blot analysis of the expression of CAF markers, including FAP, α-SMA, vimentin, and S100A4 in isolated primary fibroblasts. (C-E) CCK-8 (C), transwell migration (D), and wound healing assays (E) used to determine the proliferative and migratory capacities of RKO and LoVo cells cultured in CAF-CM or NF-CM. Scale bars, 75 μm (D), 200 μm (E). (F) Quantification of relative STK25 levels normalized to β-actin in Figure 1A. (G) Quantification of relative protein levels of CAF activation markers normalized to β-actin in Figure 1E. (H) Immunofluorescence staining showing the expression of α-SMA, FAP, and S100A4 in CAFs after co-culture with STK25-knockdown LoVo cells or STK25-overexpressing SW480 cells. Scale bars, 50 μm. One-way ANOVA was used for statistical analysis. *$p<0.05$; **$p<0.01$; ***$p<0.001$.

Figure S2 related to Figure 3

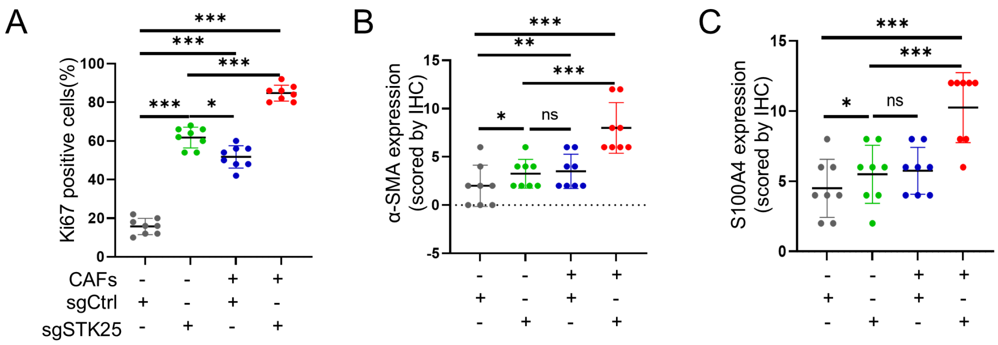

Figure S2 related to Figure 3. Quantification of IHC staining in Figure 3G. (A-C) Quantification of IHC staining of Ki67, α-SMA, and S100A4 in Figure 3G. One-way ANOVA were used for statistical analysis. *p<0.05, ***p<0.001; ns, not significant.

Figure S3 related to Figure 4

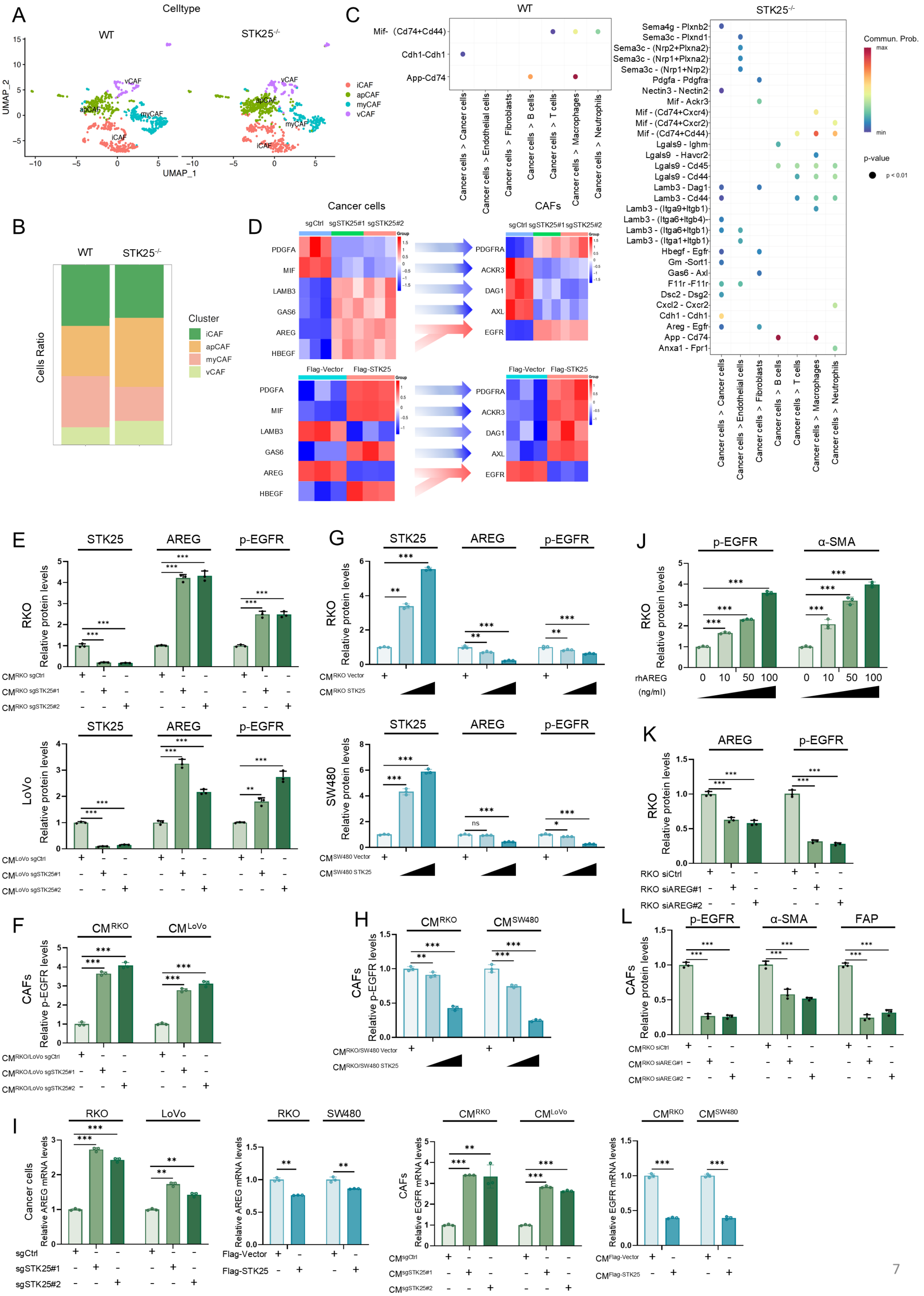

Figure S3 related to Figure 4. Validation of the AREG/EGFR signaling in STK25-regulated tumor cell-CAF communication. (A, B) Subpopulation analysis of CAFs in tumor tissues from WT and STK25$^{-/-}$ mice using scRNA-seq data from GSE277814. (C) CellChat analysis used to predict enriched ligand-receptor pairs between cancer cells and other cells in the WT and STK25$^{-/-}$ groups. (D) STK25-knockdown or STK25-overexpressing CRC cells were co-cultured with CAFs. qRT-PCR was used to determine the mRNA levels of ligand and receptor genes in CRC cells and CAFs, respectively. (E-H) Quantification of relative protein levels in Figure 4H (E), Figure 4I (F), Figure 4J (G), and Figure 4K (H). (I) qRT-PCR analysis of AREG mRNA levels in STK25-knockdown or STK25-overexpressing CRC cells and EGFR mRNA levels in co-cultured CAFs. (J-L) Quantification of relative protein levels in Figure 4L (J), Figure 4O (K), and Figure 4P (L). The protein levels were normalized to β-actin, and the levels of pEGFR were normalized to total EGFR. Two-tailed Student's t-test and one-way ANOVA were used for statistical analysis. *p<0.05, **p<0.01, ***p<0.001; ns, not significant.

Figure S4 related to Figure 5

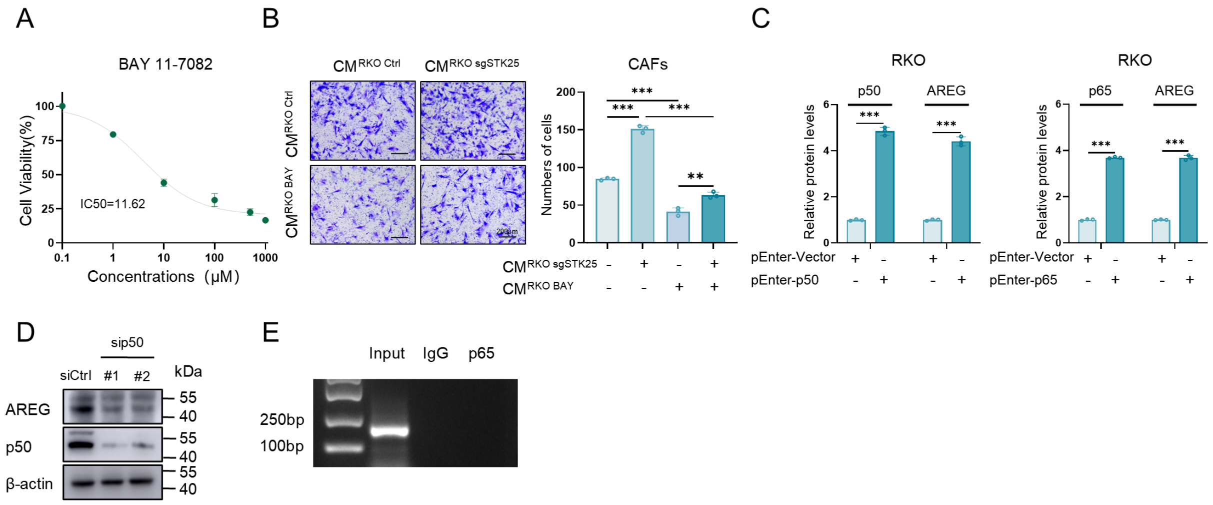

Figure S4 related to Figure 5. The NF-κB pathway was involved in STK25-mediated regulation of AREG expression. (A) RKO cells were treated with different concentrations of BAY 11-7082. Dose–response curves were plotted to determine IC50 values. (B) Transwell assays to determine the migration of CAFs co-cultured with CM from sgSTK25 CRC cells pretreated with 5 μM BAY 11-7082. Graphs representing the quantification of migrated cells. Scale bars, 200 μm. (C) Quantification of relative protein levels of p50, p65, and AREG normalized to β-actin in Figure 5E. (D) The expression of AREG and p50 in p50-knockdown CRC cells. (E) ChIP assays showed no specific binding of p65 to the AREG promoter region. Data are presented as the mean ± SD of at least three independent experiments. A two-tailed Student's t-test was used for statistical analysis. ***$p < 0.001$.

Figure S5 related to Figure 6

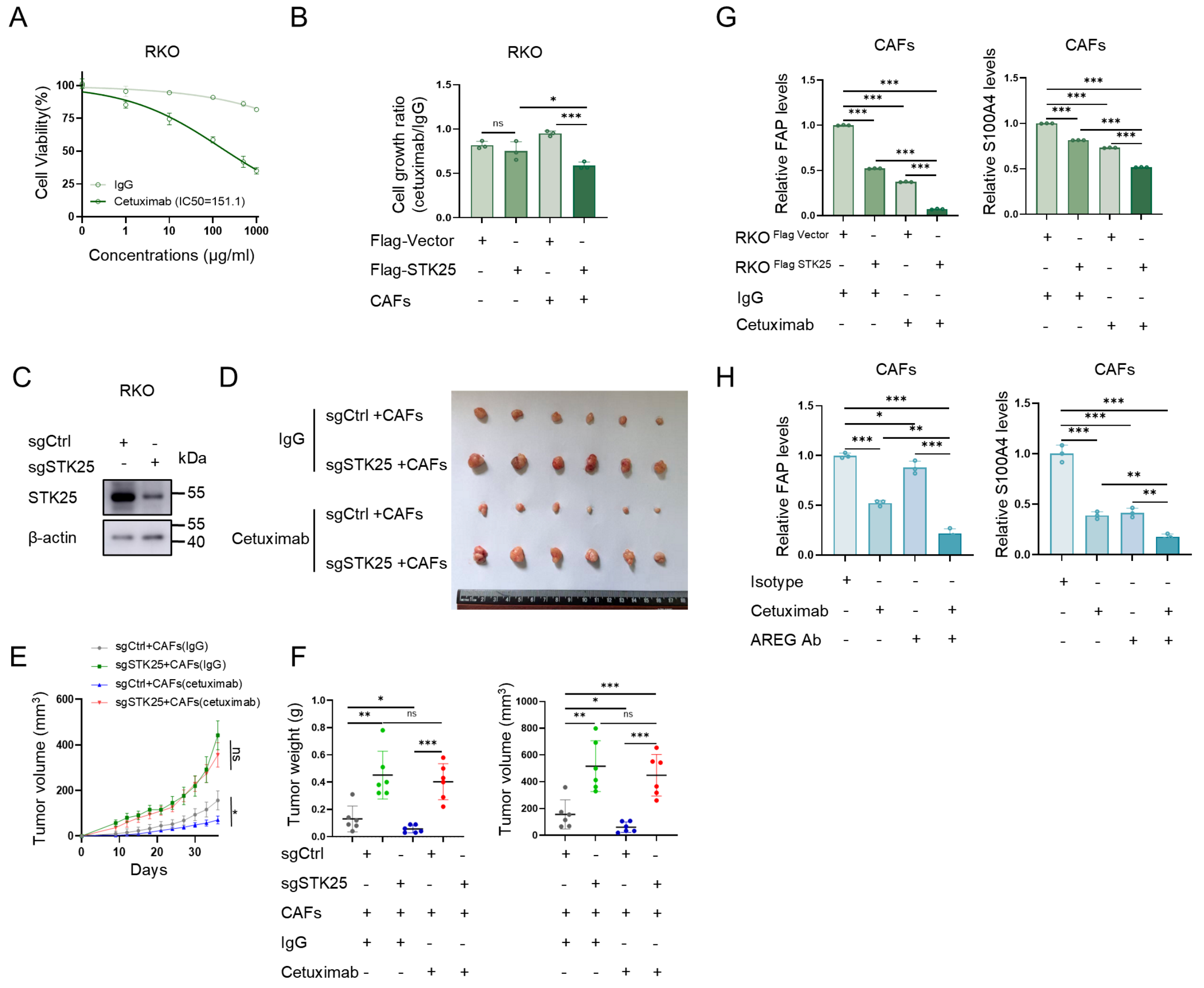

Figure S5 related to Figure 6. The association between STK25 expression and the sensitivity of cetuximab treatment. (A) RKO cells were treated with different concentrations of cetuximab, nonspecific IgG served as a control. Dose–response curves were plotted to determine IC50 values. (B) Graphs showed another representation of Figure 6A. The cell viability of cetuximab-treated cultures is shown relative to their IgG-treated controls. (C) Western blot analysis verified the knockdown efficiency of STK25 in RKO cells. (D) Nude mice subcutaneously injected with sgSTK25 CRC cells mixed with CAFs were intraperitoneally treated with 1 mg cetuximab or IgG (n = 6 per group). Images of tumor tissues isolated from mice in different treatment groups were displayed. (E) Tumor volume was measured every 3 days, and the tumor growth curves were plotted over time. (F) Tumor weight and volume of mice were recorded when mice were sacrificed at day 40. (G, H)

Quantification of relative protein levels normalized to β-actin in Figure 6E, F. Data are presented as the mean ± SD of at least three independent experiments. One-way ANOVA and two-way ANOVA were used for statistical analysis. $^{*}p < 0.05$, $^{**}p < 0.01$, $^{***}p < 0.001$; ns, not significant.

Figure S6 related to Figure 7

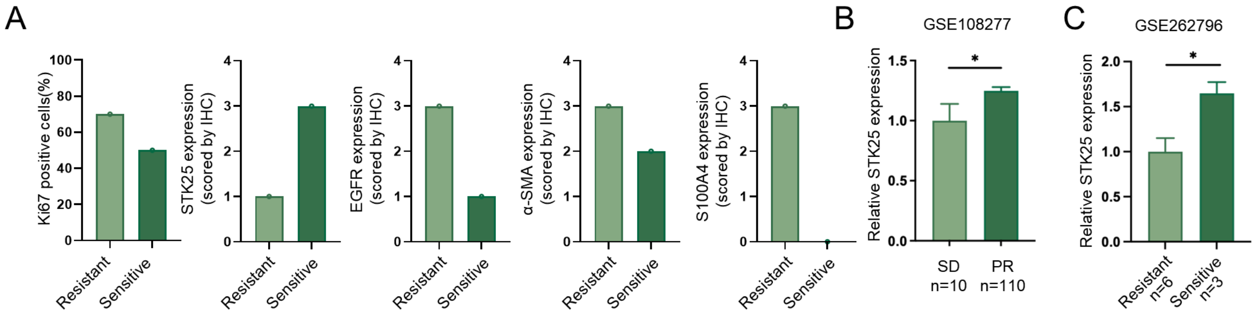

Figure S6 related to Figure 7. Validation of the correlation between STK25 expression and cetuximab sensitivity using the GEO database. (A) Quantification of IHC staining of Ki67, STK25, EGFR, α-SMA, and S100A4 in Figure 7E. A two-tailed Student's t-test was used for statistical analysis. (B) The expression of STK25 in mCRC patient-derived xenografts (PDXs) from the partial response (PR) subgroup and stable disease (SD) subgroup in the analysis of GSE108277 dataset. (C) STK25 expression in cetuximab-sensitive and cetuximab-resistant LIM1215 cells from the GSE 262796 dataset. *$p < 0.05$.

Supplementary Tables:

Supplementary Table S1: Clinical information for 4 CRC samples, related to Figure 7A

Sample	Gender	Age	Tumor location	T Stage	N Stage	M Stage	TNM stage	Histological Type	Differentiation grade	pMMR/dMMR	Preoperative treatment
Patient 1	M	69	Rectum	pT3	N0	M0	IIA	Adenocarcinoma	Middle	pMMR	No
Patient 2	M	62	Sigmoid colon	pT4a	N2b	M1	IVA	Adenocarcinoma	Middle	pMMR	No
Patient 3	F	69	Ascending colon	pT3	N0	M0	IIA	Adenocarcinoma	Middle	pMMR	No
Patient 4	M	59	Descending Colon	pT4a	N1a	M0	IIIB	Adenocarcinoma	Low	pMMR	No

Supplementary Table S2: Clinical information for 11 CRC samples, related to Figure 7B

Sample	Gender	Age	Tumor location	T Stage	N Stage	M Stage	TNM stage	Histological Type	Differentiation grade	pMMR/dMMR	Preoperative treatment
Patient 1	M	60	Hepatic flexure	pT3	N0	M0	IIA	Adenocarcinoma	Low	pMMR	No
Patient 2	M	77	Transverse colon	pT4a	N1	M0	IIIB	Adenocarcinoma	Middle	pMMR	No
Patient 3	M	81	Hepatic flexure	pT4b	N0	M0	IIB	Adenocarcinoma	Middle	pMMR	No
Patient 4	M	55	Sigmoid colon	pT2	N0	M0	I	Adenocarcinoma	Middle	pMMR	No
Patient 5	F	52	Sigmoid colon	pT4a	N0	M0	IIA	Adenocarcinoma	Middle	pMMR	No
Patient 6	M	73	Hepatic flexure	pT4a	N0	M0	IIA	Adenocarcinoma	Low	pMMR	No
Patient 7	M	54	Hepatic flexure	pT4b	N0	M0	IIB	Adenocarcinoma	Low	pMMR	No
Patient 8	M	51	Hepatic flexure	pT4a	N1	M1a	IVA	Adenocarcinoma	Middle	pMMR	No
Patient 9	F	33	Transverse colon	pT4a	N0	M0	IIA	Adenocarcinoma	Middle	pMMR	No
Patient 10	F	69	Ascending colon	pT3	N0	M0	IIA	Adenocarcinoma	Middle	pMMR	No
Patient 11	M	59	Descending colon	pT4a	N1a	M0	IIIB	Adenocarcinoma	Low	pMMR	No

Supplementary Table S3: Clinicopathological characteristics of two CRC patients who received cetuximab therapy, related to Figure 7E, F.

Sample	Gender	Age	Tumor location	T Stage	N Stage	M Stage	TNM stage	Histological Type	pMMR/dMMR	Response to cetuximab
Patient 1	M	57	Descending colon	pT3	N2b	M1	IV	Adenocarcinoma	pMMR	Sensitive
Patient 2	F	60	Rectum	pT3	N1b	M1	IV	Adenocarcinoma	pMMR	Resistant

Supplementary Table S4: Clinical information for 2 CRC samples, related to Figure 7G

Sample	Gender	Age	Tumor location	T Stage	N Stage	M Stage	TNM stage	Histological Type	Differentiation grade	pMMR/dMMR	Preoperative treatment
Patient 1	M	69	Rectum	pT2	N0	M0	I	Adenocarcinoma	Middle	pMMR	No
Patient 2	M	37	Rectum	pT3	N0	M0	IIA	Adenocarcinoma	Middle	pMMR	No

Supplementary Table S5. sgRNA and siRNA sequences

Application	Gene name	Target sequence (5’-3’)	Species
sgRNA	STK25 #1	TCATCGACCGCTATAAGCGC	Human
sgRNA	STK25 #2	GGGGATCACAGCCATCGAGC	Human
siRNA	AREG #1	GGAGCCGACTATGACTACT	Human
siRNA	AREG #2	CAGGAAATATGAAGGAGAA	Human
siRNA	p50 #1	GCACCTAGCTGCCAAAGAA	Human
siRNA	p50 #2	GATCCTTCTTTGACTCATA	Human

Supplementary Table S6. Relevant primer sequences.

Gene	Primer sequences		Species
STK25	Forward	5′- CTTGCTCTTGGGTCTCTGGAATC -3′	Human
	Reverse	5′- CTTAGGAATGTCTCGGCAGATCC -3′	Human
PDGFA	Forward	5′-GGACTCATGACCACAGTCCATG -3′	Human
	Reverse	5′- CAGGGATGATGTTCTGGAGAGC -3′	Human
PDGFRA	Forward	5′- CTTACCTGTAAACAGAGAGACAC- 3′	Human
	Reverse	5′- TTGCGCCGGTTTTTATCGATTGT -3′	Human
MIF	Forward	5′- ACATGATTCAGCCACAGATACC -3′	Human
	Reverse	5′- GCATAGATGTCAGCACGTTTG -3′	Human
ACKR3	Forward	5′- CGTCCATCTTGCCATTCACG -3′	Human
	Reverse	5′-CTCAAGCTCATCTAATCGTCCTG -3′	Human
LAMB3	Forward	5′- TGGACGCAGGTTCTCCAAAC -3′	Human
	Reverse	5′- CCGGCTCGCAGTAGGTAAC -3′	Human
DAG1	Forward	5′- ACCCATCGCTCTACCCGGCCCT -3′	Human
	Reverse	5′- ATCGGCTCCCGGCTCCGAGAG -3′	Human
HBEGF	Forward	5′- TCTCGGAGCCGGGAGCCGAT -3′	Human
	Reverse	5′- CGAGCGCCGGTTTCTGGCC -3′	Human
EGFR	Forward	5′- TCTCGGAGCCGGGAGCCGAT -3′	Human
	Reverse	5′- CGAGCGCCGGTTTCTGGCC -3′	Human
GAS6	Forward	5′- CGTCCATCTTGCCATTCACG -3′	Human

	Reverse	5′-CTCAAGCTCATCTAATCGTCCTG -3′	Human
AXL	Forward	5′- TGGACGCAGGTTCTCCAAAC -3′	Human
	Reverse	5′- CCGGCTCGCAGTAGGTAAC -3′	Human
AREG	Forward	5′- ACCCATCGCTCTACCCGGCCCT -3′	Human
	Reverse	5′- ATCGGCTCCCGGCTCCGAGAG -3′	Human
FAP	Forward	5′-CTACCCAAAGGCTGGAGCTAA-3′	Human
	Reverse	5′-ACAGGACCGAAACATTCTGG-3′	Human
α-SMA	Forward	5′-TATCCCCGGGACTAAGACGGG-3′	Human
	Reverse	5′-CAGAGCCCAGAGCCATTGTC-3′	Human
S100A4	Forward	5′-GGTGTCCACCTTCCACAAGT-3′	Human
	Reverse	5′-TGTTGCTGTCCAAGTTGCTC-3′	Human
p50	Forward	5′-AACAGAGAGGATTTCGTTTCCG-3′	Human
	Reverse	5′-TTTGACCTGAGGGTAAGACTTCT-3′	Human
p65	Forward	5′-ATGTGGAGATCATTGAGCAGC-3′	Human
	Reverse	5′-CCTGGTCCTGTGTAGCCATT-3′	Human
GAPDH	Forward	5′-GGACTCATGACCACAGTCCATG-3′	Human
	Reverse	5′-CAGGGATGATGTTCTGGAGAGC-3′	Human
